# No effect of ‘watching eyes’: An attempted replication and extension investigating individual differences

**DOI:** 10.1371/journal.pone.0255531

**Published:** 2021-10-06

**Authors:** Amanda Rotella, Adam Maxwell Sparks, Sandeep Mishra, Pat Barclay

**Affiliations:** 1 Department of Psychology, Kingston University, Kingston-Upon-Thames, United Kingdom; 2 Department of Psychology, University of Guelph, Guelph, Ontario, Canada; 3 Department of Management, University of Guelph, Guelph, Ontario, Canada; Middlesex University, UNITED KINGDOM

## Abstract

Some evidence suggests that people behave more cooperatively and generously when observed or in the presence of images of eyes (termed the ‘watching eyes’ effect). Eye images are thought to trigger feelings of observation, which in turn motivate people to behave more cooperatively to earn a good reputation. However, several recent studies have failed to find evidence of the eyes effect. One possibility is that inconsistent evidence in support of the eyes effect is a product of individual differences in sensitivity or susceptibility to the cue. In fact, some evidence suggests that people who are generally more prosocial are less susceptible to situation-specific reputation-based cues of observation. In this paper, we sought to (1) replicate the eyes effect, (2) replicate the past finding that people who are dispositionally less prosocial are more responsive to observation than people who are more dispositionally more prosocial, and (3) determine if this effect extends to the watching eyes effect. Results from a pre-registered study showed that people did not give more money in a dictator game when decisions were made public or in the presence of eye images, even though participants felt more observed when decisions were public. That is, we failed to replicate the eyes effect and observation effect. An initial, but underpowered, interaction model suggests that egoists give less than prosocials in private, but not public, conditions. This suggests a direction for future research investigating if and how individual differences in prosociality influence observation effects.

## Introduction

People often act prosocially, behaving in ways that benefit others, even at cost to themselves. Prosocial behaviours are widely documented, are often directed towards strangers, and are performed in anonymous settings [1]. Although cooperation in contexts with no opportunity to be repaid may appear puzzling, this can be functionally accounted for by indirect reciprocity, under conditions of uncertainty regarding reputational consequences.

Someone who has established a good reputation is often rewarded by others through preferential helping and being chosen as friends or cooperative partners. These long-term benefits, which are attributable to a good reputation, often outweigh the short-term costs of behaving cooperatively. Functional analyses show this can be an evolutionary stable strategy [2–6], and evidence that people use this strategy has been demonstrated in several lines of research. People behave more cooperatively towards others with good reputations and are more likely to choose partners for future interactions that good reputations in a partner-choice scenario [7–11]. Additionally, when people have the opportunity to earn a good reputation (i.e., when they are being watched or when others will be informed of their behaviours) they behave more cooperatively [6–16]. These findings demonstrate that people sensitive to cues of observation calibrate their behaviours to earn a good reputation [17].

Over the past 15 years, research has found that even invalid cues of observation, such as images of eyes (or eyespots; “the watching eyes effect”), can be enough to influence behaviour in the absence of actual observation [18–20]. Eyespots are thought to trigger feelings of observation, which recruit a subconscious reputation management system to modify behaviour. This effect has been observed in both lab experiments [20–24], and field studies [18,25–27], and across many dependent measures, including increasing generosity [22,23,28–30], condemnation of moral violations [31], and (reducing) antisocial behaviours (for review see [32]). These findings suggest that people are very sensitive to reputation-based cues; even invalid cues of observation can affect social decisions.

However, many recent studies have failed to replicate the watching eyes effect [21,24,33–38]. This evidence calls into question the replicability of the effect [38]. Towards attempting to explain variation in eyes effect results, one study found evidence for the watching eyes effect, but only if eyes are presented briefly and participants do not have time for habituation to the cue [21]. These mixed results support the general notion that the non-replications of the watching eyes effect may be in part due to yet-unmeasured moderators.

### Individual differences and reputation

Little of the above research has explicitly investigated individual differences in susceptibility to reputation-based cues. Indeed, indirect reciprocity may not be used in the same way by everyone; empirical studies have found that people who are more dispositionally prosocial are less responsive to reputation-based cues and less responsive to manipulations that enhance concern for reputation such as shame, compared to people who are less dispositionally prosocial (i.e., egoists) [39,40]]. Given that many people tend to be consistently generous (or consistently selfish) across time and situations (e.g., [41–45]), we posit that people who are more prosocial would be less influenced by positive reputation-based cues (e.g., observation, eyes effects) because they are already prosocial. In other words, they already have high levels of prosociality and have limited potential to increase prosocial behaviors beyond their baseline in response to reputation-based cues. Alternatively, people who are less prosocial, such as egoists, can receive more reputation-based benefits by increasing their prosocial behaviors beyond their baseline levels of prosociality, and have more potential to do so because their baseline levels of prosociality are lower than that of prosocials. Thus, it is possible that people who are more prosocial may be less sensitive to positive reputation-based cues, leading to inconsistent watching-eyes results. Given there are more prosocial people than selfish people (see Supplementary Material), null effects may be a result of high levels of baseline prosociality among participants.

### Goal and hypotheses

The primary goal of this study was to replicate the watching eyes effect in a high-powered sample, adhering closely to previous methodology (i.e., [23]). Additionally, we sought to determine if people who are less dispositionally prosocial (as measured by social value orientation, SVO) are more responsive to the presence or absence of invalid cues of observation (i.e., eyespots). Towards the latter goal, we sought to conduct a conceptual replication of Simpson and Willer (2008) [39] to investigate if SVO has different relationships with decision-making in public versus private conditions. We hypothesized that a person’s prosocial disposition would relate to their responsivity to reputation-based cues. We predicted people who are more prosocial would be less responsive to reputation-based cues than people who are less prosocial, and that this effect would be stronger for real cues of observation rather than invalid cues (i.e., eyespots) of observation.

To test our main hypothesis about the eyes effect, participants played a dictator game, making a decision about how to split $10 between themselves and another participant, in one of three conditions: (1) the *public* condition where their dictator game decisions would be made known to other participants, (2) the *eyes* condition where participants would be exposed to images of eyes (i.e., an invalid cue of observation) before their anonymous decision in the dictator game, and (3) a *no-eyes control* condition where participants made anonymous decisions without being exposed to reputation-based cues. To test our secondary hypothesis about SVO and reputation cues, participants completed a measure of dispositional prosociality (SVO, described below) and we used their responses to categorize them as either prosocial or egoist.

We made the following predictions:

1. We predict that people would give more in the dictator game in the *public* condition than in the *eyes* condition (given that stylized eyes are an invalid cue of observation), and would give less in the *no-eyes control* compared to both other conditions. That is, we predict both an observation effect and a “watching eyes” effect (i.e., an ‘invalid’ observation effect’).
2. People who are more dispositionally prosocial (i.e., SVO prosocials) will be less susceptible to situational reputation-based effects. We predict that SVO prosocials would show no differences in dictator game allocations in the *public*, *eyes*, or *no-eyes* control conditions.
3. People who are less dispositionally prosocial and prefer to maximize their own income (i.e., SVO egoists) should be more susceptible to reputation-based effects. We predict SVO egoists would give more in the dictator game in the *public* and *eyes* conditions than in the *no-eyes control*.

## Methods

Our hypotheses, predictions, methods, and analysis plan were pre-registered at https://osf.io/qse6fand R scripts and data are available at https://osf.io/bndx6/. This study was approved through the Research Ethics Boards at the University of Guelph (#15AU015) and the University of Regina (#2016–103). Signed consent forms were obtained from participants.

### Participants

A total of 356 students at the Universities of Guelph and Regina participated in this study in exchange for course credit plus game earnings. One participant did not have a dictator game response and was excluded from the study, leaving 355 students (23% male, 60% female, 17% missing; *M*_*age*_ = 19.47 *SD*_*age*_ = 3.45) in the total sample. We excluded 10 participants whose SVO responses did not meet classification criteria for the Triple-Dominance Measure of SVO (i.e., had fewer than 6 consistent choices), and another 160 participants whose SVO responses were unknown because we were unable to link our pre-term survey with in-lab responses (see below), leaving 189 participants with SVO data (118 prosocials, 71 egoists). Our total sample exceeded our minimum pre-registered sample of 200.

### Procedures

#### Pre-term survey

At the beginning of the term, students completed the Triple-Dominance Measure of SVO [46] as part of a pre-term survey for psychology students. Participants completed nine SVO questions assessing participants’ preferences of point distributions between themselves and a hypothetical ‘other’ [46]. All questions included three potential choices: a prosocial choice where participants prefer an equal distribution of points for themselves and the other (e.g., 500 self, 500 other), an egoistic choice where participants could maximize their earnings while disregarding the earnings of the other (e.g., 550 self, 300 other), and a competitor choice which maximizes the difference between themselves and the other (e.g., 500 self, 100 other). Participants were categorized as “prosocial”, “egoist”, or “competitor” if they selected six or more consistent choices.

As part of the pre-term survey, participants also filled out a unique participant-generated code to link this data with data provided in the lab without compromising anonymity (see supplementary material). This measure was decoupled from the lab experiment to avoid priming and spillover effects from the experimental procedures (i.e., the method of measuring SVO could influence the dependent outcome, or vice versa).

Participant codes for the pre-term survey could not be matched with the code provided in-lab for 160 participants. The majority of these were from the first term of data collection at the University of Guelph (*n* = 116) because there was an error in the survey administration. These participants were not included in analyses involving individual differences in SVO; however, these participants were included in the analyses investigating how observation and eye images influence prosociality.

#### Pre-screening

The three social value orientations occur in different frequencies in populations, with prosocials as the most common (see supplement and [46,47]). Accordingly, to recruit similar numbers of each orientation, we had to selectively recruit egoists. We could not use the pre-term survey to do so because results from the survey were not accessible until the completion of the term. Consequently, we pre-screened in-lab participants with three items from the Triple Dominance Measure to approximate SVO. Participants completed the remainder of the experiment if they had three consistent choices as prosocial (i.e., indicated they preferred equal distributions of points between themselves and a hypothetical other, such as 500 for self and 500 for other) or as an egoist (i.e., had three consistent choices of maximizing their own outcome, such as 560 for self and 300 for other).

To avoid biasing the in-lab experimenter, an independent research assistant scheduled participants for the in-lab. Therefore, the in-lab experimenter was blind to the SVOs of the participants in each condition.

#### Experimental design

**Fig 1:**
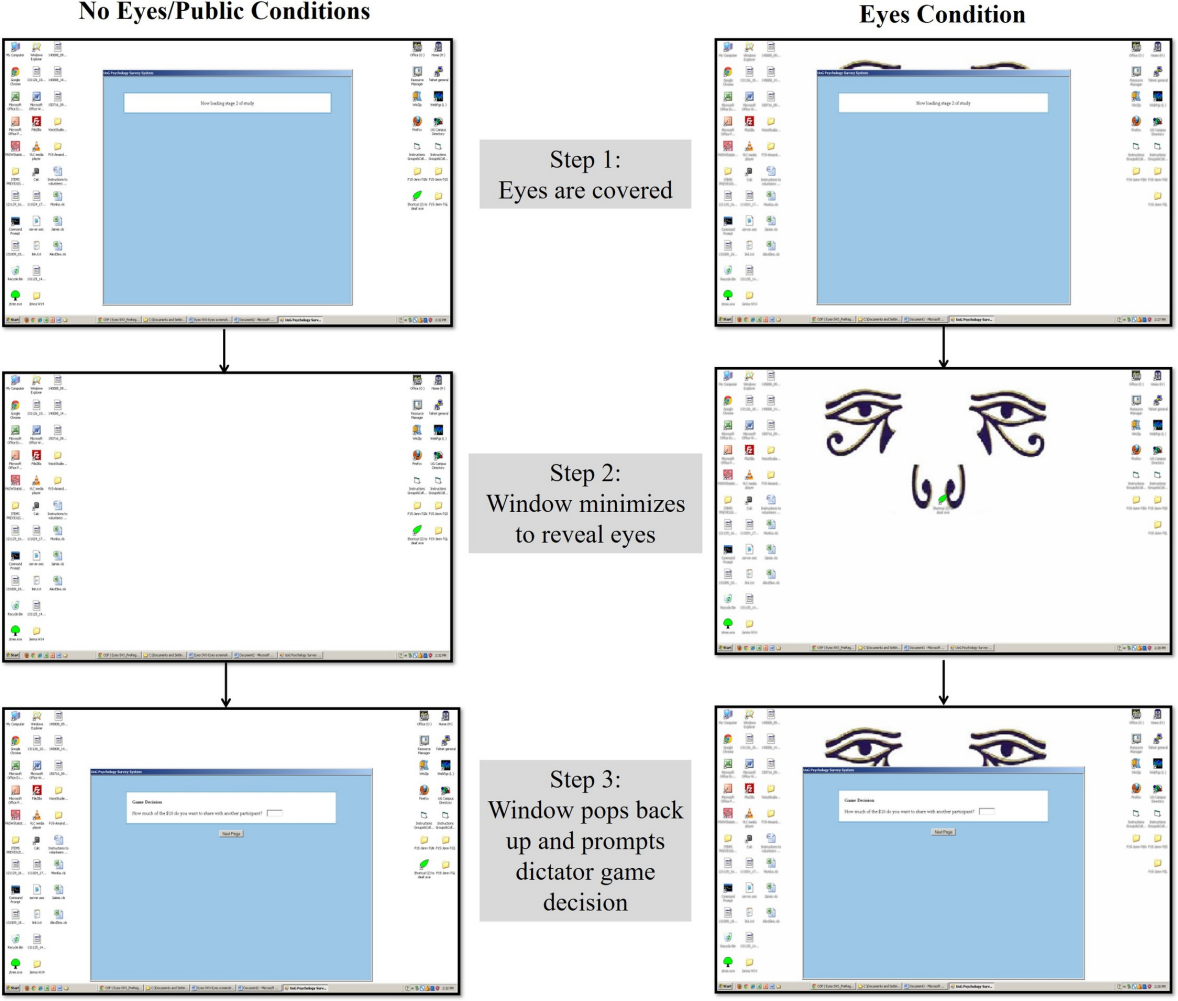
Participant view during the dictator game decision, by condition.

Methods were adapted from Sparks and Barclay (2013) [23]. Participants were randomly assigned to one of three experimental conditions: No Eyes (*n* = 113), Eyes (*n* = 117), and Public (*n* = 100). In the No Eyes and Public conditions, computer desktop backgrounds were blank, whereas in the Eyes condition the backgrounds included stylized eyespots commonly used in such studies [20,23,28,29]; see Fig 1 or Supplementary Material for a more detailed description of the program.

Students participated in groups of four to six. After arriving at the lab and providing consent, the experimenter would provide an overview of the tasks and how earnings would be distributed. Participants were told they would participate in “at least one economic game where they would decide how to allocate money between themselves and another participant, but that payment would be based on the results of one randomly selected game”. Participants then sat themselves at computers, which were out of sight from the experimenter, and began the self-guided computer program. Participants completed demographic questions, followed by specific instructions about the Dictator Game, in which they would get $10 CAD (in real currency) to divide as they wanted between themselves and another participant [20,23].

In all conditions the computer program closed after the completion of the demographic questionnaire, after which a brief message appeared for three seconds indicating that the next section was loading. Subsequently, the window re-appeared to prompt participants for a decision in the dictator game. In the Eyes condition, the program window covered the eye images during the demographic questions, but the eyes were fully revealed before the dictator game decision. Eyes were only presented briefly because long-exposure to eyes appear not to elicit “eyes effects” [23]. After the dictator game, participants proceeded to the post-experimental survey described below.

After they completed these tasks, participants left the computer room. Once all participants left the computer room, the experimenter distributed the game earnings in envelopes by the participants’ computer, and then met participants in the common area for a debriefing. Participants then re-entered the computer room to claim their earnings and left. We used a random number generator to determine if they received payment for their decision or the decision of another participant.

In the No Eyes and Eyes conditions, participants were truthfully told that their decisions would be anonymous, and not be known to the experimenter or to the other participants in the session.

In the Public condition, participants were given lanyards with a unique participant number (easily visible to all participants). Participants were informed that they would view a dry erase board displaying (i) their participant number, (ii) their decision, and (iii) the participant number and decision made by their partner (randomly assigned by the experimenter), at the end of the session. While delivering these instructions, the experimenter pointed to the blank dry erase board (which was removed in the eyes and control conditions), which was filled out and displayed at the end of the session. Thus, in the Public condition, participants were informed that they could determine everyone’s decisions and how it affected their outcome. Deception was not used in this experiment.

#### Post-experiment survey

After the experimental manipulation and the dictator game, participants completed the following scales and questions.

#### Post-experiment questionnaire

Participants completed 17 items assessing feelings and impressions of participants during the dictator game (each measured using a 7-point Likert scale). This scale was originally developed by [28] and was presented in [29]. The first eight items asked about what factors participants considered when they were deciding dictator game allocations (e.g., “I should think of the recipient” and “I will feel guilty if I don’t share an equal amount with the recipient”), the next three items asked about their concerns about the allocations (e.g., “Someone will see the amount of money I allocated and think I am a bad person”), and the final six items asked about perceptions of the experimental situation (e.g., “A situation in which other people would evaluate my behavior”).

As time filler in these methods (and providing exploratory pilot data for a separate project), participants subsequently completed the SVO slider measure [47] and a Machiavellianism scale ([48]; scales are described in Supplementary Material). Lastly, participants completed a free response to the question “What do you think was being investigated in this study?”, rated the likelihood that they would tell others about the study, and created the participant-generated ID to link their data to the pre-term survey.

### Analyses

We fit ANOVA models in R version 3.6.0 [49], using effect sizes as the basis of interpretation [50]. The *yarrr* package was used for data visualization [51], and the *data*.*table* [52], *sjstats* [53], *apaTables* [54], *lsr* [55], and *psych* [56] packages were used to compute effect sizes and their confidence intervals. Note that 90% confidence intervals were used for partial eta squared, which are equivalent to 95% confidence intervals for Cohen’s d (see Supplementary Material for justification).

## Results

### Manipulation checks

**Fig 2:**
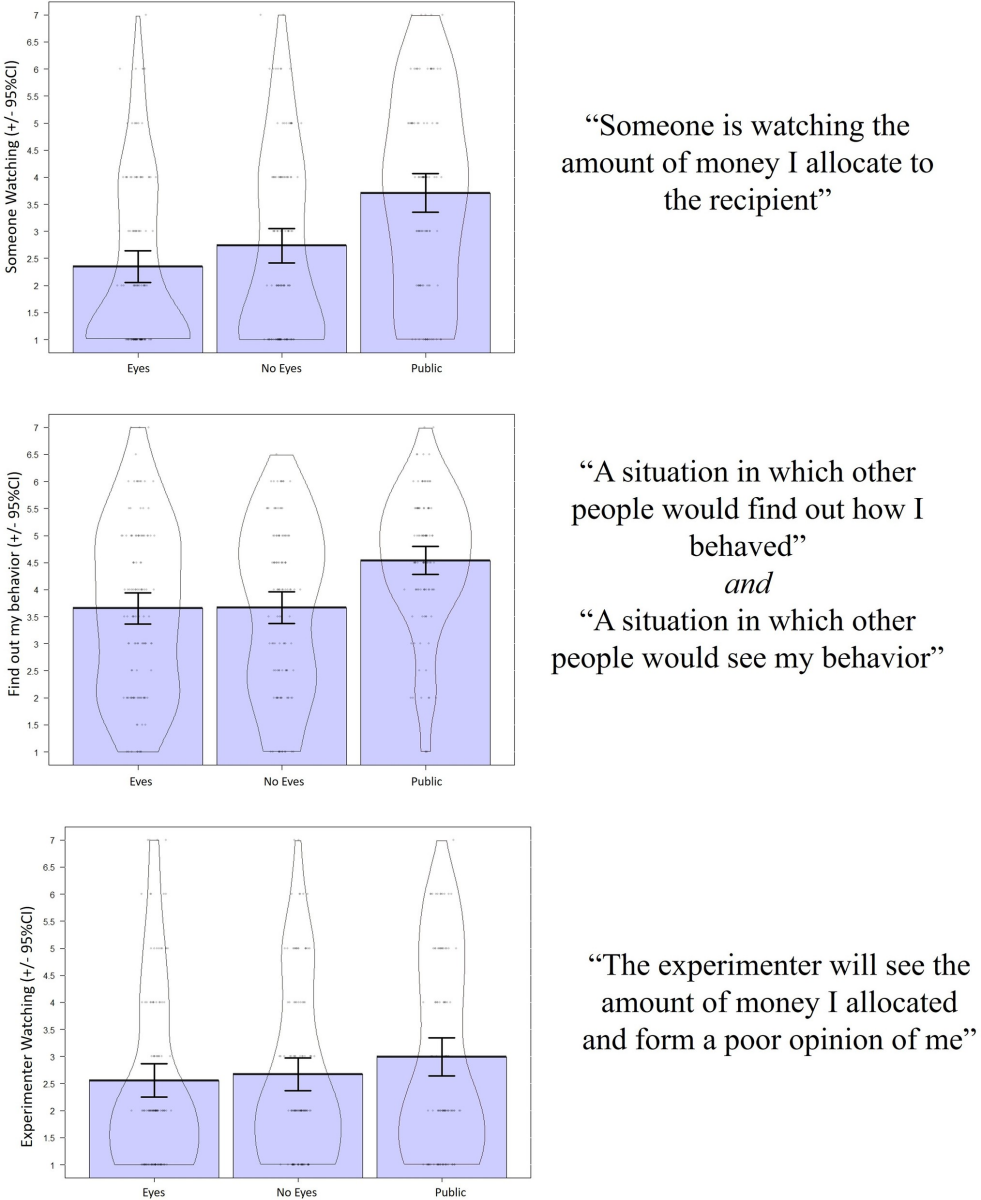
Participant agreement with post-experimental questions by condition (+/- 95% CIs), and the associated question(s) to the right. Colored areas represent traditional bar graphs. Contours are violin plots which demonstrate the distribution of responses for that condition. Dots are individual responses, with jitter.

As manipulation checks, we compared responses to four questions in the post-experimental questionnaire. Although we pre-registered inclusion of the post-experimental questionnaire, we did not pre-register an analysis plan. Thus, these manipulation checks should be considered exploratory. Figures of responses are presented in Fig 2.

To assess whether participants felt observed, participants rated agreement with the following statement “Someone is watching the amount of money I allocate to the recipient”. If our manipulations worked, participants should feel most observed in the public condition, followed by the eyes condition, in comparison to the control condition. Partially supporting this manipulation, responses on this item differed by condition, *F*(2, 356) = 16.24, *p* < .001, *η*_*p*_^*2*^ *=* .084, 90% CI [.04, .13]. There were no differences between the eyes (*M* = 2.39, *SD* = 1.66) and no eyes (*M* = 2.74, *SD* = 1.68) conditions, *t*(247.93) = -1.67, *p* = .097, *d* = 0.21, 95% CI [-0.04, 0.46]. This suggests that people did not feel more observed in the *eyes* condition compared to the control condition. Given that the effect size was smaller than in prior studies, our power to detect a small effect of the same size (d = .21) was only 0.38; accordingly, our study had a small probability of finding a statistically-significant result, even if a true effect existed. Participants felt more observed in the *public* condition (*M* = 3.64, *SD* = 1.81) than the *no eyes* condition, *t*(222.39) = -3.91, *p* < .001, *d* = 0.52, 95% CI [0.25, 0.83], and to the *eyes* condition, *t*(220.83) = -5.49, *p* < .001, *d* = 0.72, 95% CI [0.45, 0.99]. These were medium and large effects, respectively (Fig 2A). Thus, it appears that our manipulation for the public condition was successful, but not the eyes condition.

Participants also rated how much they agreed to the following statements: “A situation in which other people would find out how I behaved”, and “A situation in which other people would see my behavior”. Both questions provided similar patterns of results, and therefore were combined into a single analysis (Fig 2B). Again, we expected higher responses on these items in the public condition than the eyes condition, and both conditions would have higher ratings than the control condition, if our reputation-based manipulations were successful. Responses on this aggregate item differed by condition, *F*(2, 356) = 12.12, *p* < .001, *η*_*p*_^*2*^ *=* .069, 90% CI [.03, .11]. There were no differences between the *eyes* (*M* = 3.60, *SD* = 1.61) and *no eyes* (*M* = 3.70, *SD* = 1.55) conditions, with the effect size confidence interval including zero, *t*(247.67) = -0.50, *p* = .618, *d* = 0.06, 95% CI [-0.18, 0.31]. However, people rated higher agreement in the *public* condition (*M* = 4.54, *SD* = 1.33) than the *no eyes* condition, *t*(231.90) = -4.42, *p* < .001, *d* = 0.57, 95%CI [0.31, 0.84], and compared to the *eyes* condition, *t*(231.26) = -4.85, *p* < .001, *d* = 0.63, 95%CI [0.36, 0.90]. Both of these effects were moderate in size (Fig 2A).

These results suggest participants felt more observed by others, specifically by other participants and not the experimenter, in the *public* condition compared to the *eyes* and *no-eyes control* conditions. Interestingly, results also suggest participants in the *eyes* condition did not feel more observed during the experiment compared to those in the other conditions. This suggests that eyes did not elicit an observation effect. Alternatively, this finding may be due to the fact the observation questions were presented near the end of the survey, several minutes after participants were exposed to the eyes, which may be enough for eyes effects to dissipate [22]. People in the anonymous condition also felt somewhat observed. Additional analyses involving the post-experimental questionnaire are presented in supplementary material.

### Prediction 1: Replication of the watching eyes effect

**Fig 3:**
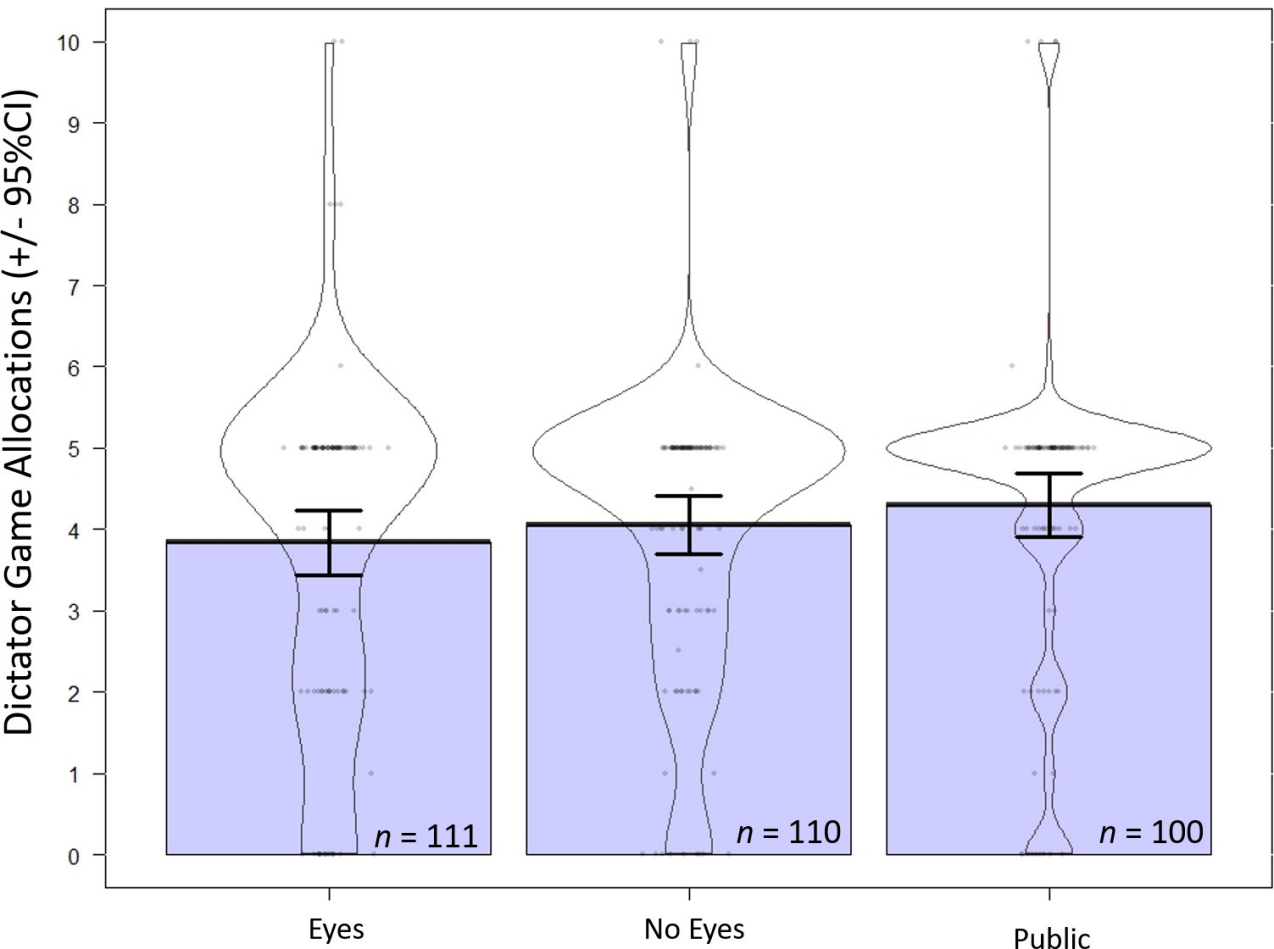
Dictator game allocations by condition (+/- 95% CIs; *N* = 355). Colored areas represent traditional bar graphs. Contours are violin plots which demonstrate the distribution of responses for that condition. Dots are individual responses, with jitter so they don’t all overlap.

We predicted that people would give more in the dictator game response to reputation-based cues, where people would give most in the public condition (real reputational cues), followed by the eyes condition (invalid cue of reputation), and least in the control condition (no reputation). To assess this, we conducted a one-way ANOVA by condition. Dictator game allocations did not differ by condition, *F*(2, 353) = 1.02, *p* = .360, *η*_*p*_^*2*^ *=* .006; Fig 3. As an exploratory analysis, we coded dictator game responses as either “gave half” (i.e., $5) or “less than half” (i.e., < $5). A chi-square test of independence indicated that results did not differ by condition, *χ*^*2*^(2) = 2.40, *p* = .302. Thus, we reject our first prediction.

### Predictions 2 and 3: Individual differences in response to reputation and eye images

**Fig 4:**
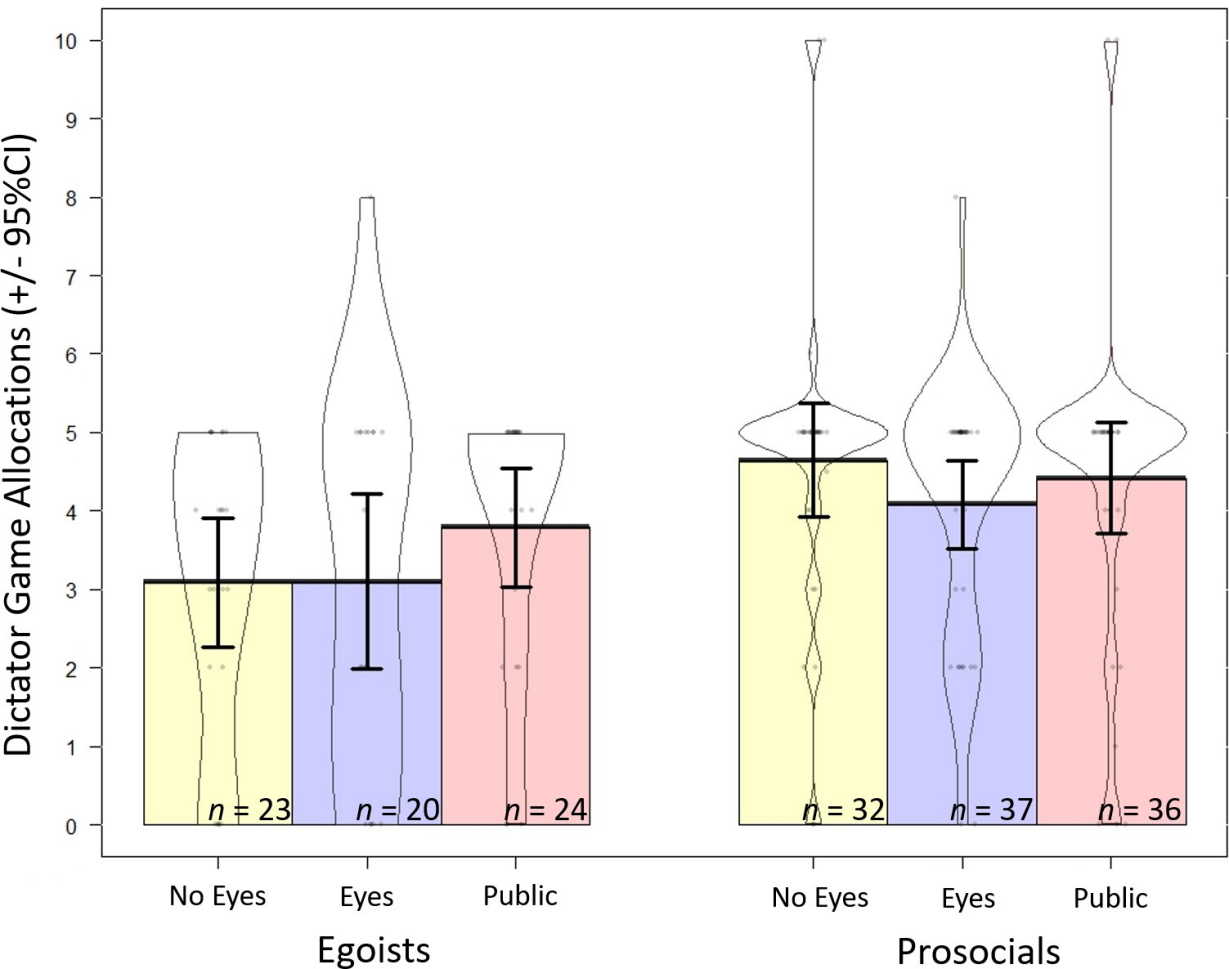
Dictator game allocations by condition and SVO (+/- 95% CIs). Colored areas represent traditional bar graphs, where egoists are in blue, and prosocials in green. Contours are violin plots which demonstrate the distribution of responses for that condition. Dots are individual responses, with jitter so they don’t all overlap.

A two-way ANOVA revealed no significant interaction between condition (eyes, no-eyes control, public) and SVO, *F*(2, 182) = 0.30, *p* = .740, *η*_*p*_^*2*^ *=* .003 (Fig 3). Again, we did not find a main effect of condition on dictator game decisions, *F*(2, 182) = 0.61, *p* = .542, *η*_*p*_^*2*^ *=* .007 (Fig 4). Given that the effect size was smaller than previous experiments (see [23]), we had limited power to detect effects. For an effect of the same size as that observed for the no eyes/public comparison (d = 0.17), power was only 0.15; accordingly, our study had a small probability of finding a statistically-significant result, even if a true effect existed. In this analysis, only SVO predicted dictator game decisions, *F*(1, 182) = 12.88, *p* < .001, *η*_*p*_^*2*^ *=* .065: we observed a medium effect of prosocials giving more (*M* = 4.33, *SD* = 1.89) than egoists (*M* = 3.29, *SD* = 2.02; Fig 4). Results were qualitatively similar when location (Regina or Guelph) was included in the model.

#### Exploratory analyses: Simplified conditions

**Fig 5:**
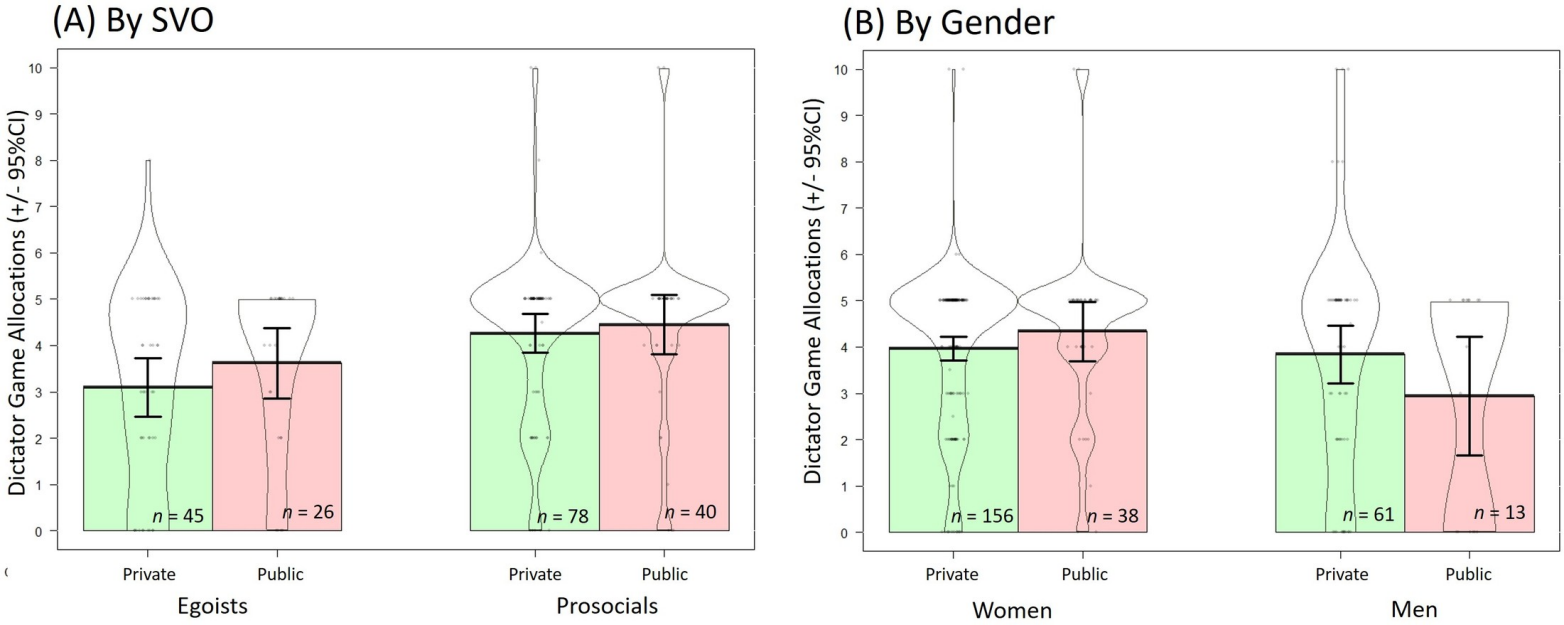
Dictator game allocations by public and private (combined eyes and control) conditions. Plot (A) is separated by SVO, where colored areas represent traditional bar graphs, where egoists are in blue, and prosocials in green. Plot (B) is separated by gender, with women in red and men and blue. Contours are violin plots which demonstrate the distribution of responses for that condition (+/- 95% confidence intervals). Dots are individual responses, with jitter so they don’t all overlap.

To simplify comparisons, we combined the *eyes* and *no-eyes control* conditions into a single *private* condition to determine if individual differences in SVO influenced dictator game allocations when decisions were anonymous (i.e., private) or not (i.e., public). This analysis was not pre-registered, and can be considered exploratory, or as a replication of Simpson & Willer (2008; [39]). Again, we did not find an interaction between condition and SVO, *F*(1, 184) = 0.30, *p* = .582, *η*_*p*_^*2*^ *=* .002, 90% CI [.00, .025], or an effect of condition, *F*(1, 184) = 0.90, *p* = .345, *η*_*p*_^*2*^ *=* .005, 90% CI [.00, .035]. There was a medium effect of SVO on cooperative decisions, *F*(1, 184) = 21.84, *p* < .001, *η*_*p*_^*2*^ *=* .065, 90% CI [.019, .129]. See Fig 5A. An analysis examining effect of simplified condition on game decisions (without SVO) revealed similar results, *F*(1, 354) = 1.98, *p* = .160, *η*_*p*_^*2*^ *=* .006, indicating non-replication of the watching eyes effect.

Inspection of Fig 3 suggested a difference in the *private* condition, which prompted exploration of pairwise comparisons. We found prosocials gave more than egoists in the private condition (prosocials: *M* = 4.26, *SD* = 1.85; egoists: *M* = 3.09, *SD* = 2.09), *t*(80.56) = -3.10, *p* = .003, *d* = 0.60, 95% CI [.21, 0.99], but not in the public condition (prosocials: *M* = 4.46, *SD* = 2.0; egoists: *M* = 3.62, *SD* = 1.88), *t*(55.95) = -1.72, *p* = .091, *d* = 0.43, 95% CI [-0.08, 0.93], which suggests that SVO egoists give similarly to prosocials in the public but not private conditions, where they give less than prosocials. When comparing dictator game allocations among egoists in public and private conditions we did not find any differences, *t*(57.16) = -1.08, *p* = .284, *d* = 0.28, 95CI[-0.23, 0.74]), however this analysis was underpowered with only 71 egoists in the analysis. As such, these results provide only weak support for our predictions (2 and 3) that egoists and prosocials respond differently to reputation-based cues.

#### Additional exploratory analyses: Gender

To identify any gender effects, we conducted a two-way ANOVA (public/private, male/female) to compare the behavior of men and women in private compared to public conditions. We did not find an effect of gender (*F*(1, 294) = 2.00, *p* = .159, *η*_*p*_^*2*^ *=* .007), nor condition (*F*(1, 294) = 0.00, *p* = .987, *η*_*p*_^*2*^ *=* .000). There was a marginal interaction of gender and condition, *F*(1, 294) = 3.67, *p* = .056, *η*_*p*_^*2*^ *=* .012, where men gave marginally less money than women in the public condition (*t*(23.62) = 2.06, *p* = .051, *d* = 0.66, 95% CI [.01, 1.31]), but not the private condition, *t*(86.34) = 0.37, *p* = .711, *d* = 0.06, 95% CI -0.23, 0.36] (see Fig 5B). These results should be interpreted with caution because there were few male participants in this study.

## Discussion

This study examined if people were more prosocial in *public*, under *“watching eyes”*, or in a control condition with *no-eyes*. We failed to replicate the previously reported eyes and observation effects. Our results suggest that prosocial disposition (as measured by social value orientation) relates to responses to reputational incentives, where SVO prosocials gave similar amounts in both public and private conditions, but SVO egoists give less than prosocials in private conditions. Only SVO was a consistent predictor of dictator game donations, with prosocials giving more than egoists. Below we discuss each of these results and study limitations.

### Failed replications: Observation and ‘watching eyes’ effects

Our manipulation check found that participants felt more observed in the public condition compared to both the eyes and no eyes conditions, suggesting that our public manipulation worked. Despite this, participants did not give more in the dictator game in the public condition compared to the eyes and control conditions. That is, we did not find an observation effect. This result was surprising, given that many prior studies suggests that people are more generous when they are being watched [6–9,11–16,39].

Based on the effect size for watching eyes in a prior study using similar methodology (i.e., short exposure to eyespots; Cohen’s f of .21 [23]), our sample of 355 participants would have given us 95% power to detect the eyes effect and observation effect. Despite this, we did not replicate the canonical “watching eyes” effect. Thus, our first prediction was not supported.

Our result is consistent with several recent failed replications [33–39]. Notably, a recent meta-analysis argues that eyes effects are effective at reducing antisocial behavior, with the speculation that images of eyes may be more effective at reducing bad behaviours than increasing good ones [32]. Watching eyes may not be particularly effective at increasing prosocial behaviours.

### Reputation and social value orientation

In our pre-registered analysis, egoists did not give less than prosocials across all three conditions. However, we conducted an exploratory analysis where we combined the no eyes control condition and eyes condition into a single private condition to replicate the analyses in a prior study [39]. Although the overall analysis did not reach statistical significance, egoists gave less than prosocials in private conditions, but not in public conditions. This finding is consistent with the prior study [39], where proselfs (egoists and competitors combined) contributed less in private conditions, whereas prosocials did not. This result suggests that egoists give less than prosocials in a dictator game when anonymous. When comparing dictator game allocations among egoists in public and private conditions we did not find any differences. Given that egoists give less than prosocials in anonymous conditions, this suggests that the strategic motives of egoists are different than that of prosocials. Notably, this analysis was underpowered and we cannot draw definitive conclusions whether SVO relates to responses to observation.

Although we had a larger sample in this study compared to Simpson and Willer (2008; [39]), they used a decision with consequences as their primary dependent measure (i.e., participants were informed that a third party could see their decision and use it to inform a subsequent decision). Their manipulation was likely stronger than a decision without consequences, as employed in the present study. It is worth noting, however, that our study was underpowered to find this effect; we could not match the SVOs of approximately 44% of participants due to an error in survey administration. Nevertheless, this is the third study suggesting that SVO may relate to responses to reputation-relevant stimuli and emotions; future studies should continue to investigate the role of individual differences in reputation-based responses.

Notably, our results are suggestive of gender effects in response to reputation-based cues. Researchers have previously proposed gender differences in prosociality [57,58], though see meta-analysis in [59], and recent research finds that people expect women to be more prosocial than men [58]. These findings suggest that there may be gender differences in reputational costs/benefits for acting prosocial in public contexts, which should be further investigated.

### Limitations

The most notable limitation in this study is our sample size. Although our sample was sufficient to replicate the observation and eyes effects, given prior samples (we had 95% power), we could not match the SVOs to their in-lab data of for a large proportion of participants, limiting our ability to draw conclusions about how SVO influences participants responses to reputation-based cues. These results should therefore be interpreted with caution. Despite this limitation, our sample size is much larger than those included in the original study (189 participants, compared to 89 and 70 in two studies [39]). This larger sample can provide a more accurate effect size to estimate power and sample sizes for future studies.

Another possible limitation to our study is that participants gave close to ceiling in the dictator game (i.e., $5) in all conditions (overall *M* = 4.06, *SD* = 2.00; all medians = 5), which may have limited our ability to find an observation effect. In fact, 62.4% of participants gave at ceiling in the public condition, and 53.6% in the private condition. However, prior research on eyes effects with a dictator game also found high allocations in the control condition (i.e., $4 out of $10) and found that images of watching eyes increased dictator game allocations beyond $4 [23]. Given that our study used similar methodology as Sparks and Barclay (2013) [23], we can conclude that we failed to replicate the eyes effect in this study. Participants did not report feeling more observed in the presence of eyes and did not give more money in a dictator game when images of eyes were present compared to the control condition. We also failed to replicate an observation effect, despite people feeling more observed in the public condition compared to the control condition, which suggests that people may not always increase cooperation when there are reputational incentives. Notably, many studies investigating observation and eyes effects do not include manipulation checks to confirm if participants feel observed. Future research could investigate when and why we would expect observation effects to occur and should include manipulation checks to confirm the experimental manipulation.

Additionally, people in our anonymous control condition (i.e., no eyes control) reported feeling somewhat observed, likely because they were in a lab environment, where there are some cues of observation such as the presence of other participants and the experimenter [59,60]. Although participants in the public condition reported feeling more observed than those in the control and eyes conditions, their scores were close to the midpoint of the scale, which suggests that participants in the public condition didn’t feel particularly observed. Notably, perceptions of observability were not correlated with dictator game allocations (see supplementary material).

A recent meta-analysis found that decisions with consequences—where participants expected their behaviours to influence how others will respond to them within the experimental protocol—produced larger observation effects on economic game allocations than decisions without consequence (*r*s of 0.25 and 0.12 respectively; [14]). The dictator game decision in this experiment was a decision without consequence, which may have limited the strength of our manipulation. However, studies using similar methodologies in small group sessions (as in this study) have reported eyes effects [20,23]. We also note that the ‘revelation moment’ differed between the eyes condition and public condition. In the eyes condition, reputational cues (eyes) were revealed right before the dictator game decision, whereas in the public condition participants were told more in advance that others would see their decisions, but the decisions were only made known to others after all decisions were made. Although both of these conditions are comparable to our control condition, these methodological differences may alter participants’ response patterns and should be considered when designing future studies.

Moreover, there are methodological similarities between SVO measures and the dictator game, where both measures ask participants to divide resources. In the present experiment, a key difference is that the dictator game is incentivized and continuous, while the SVO task is a series of hypothetical forced-choice scenarios. A conceptual replication with another measure of prosocial (or antisocial) behavior is needed to determine the generalizability of how SVO relates to prosocial behaviors.

Given the limitations outlined above, future research should investigate individual differences in observation and ‘watching eyes’ effects using dependent measures with greater reputational benefits or costs (see [32]). Moreover, future studies could use the SVO slider measure [47], as opposed to the triple-dominance measure employed in the present study. The SVO slider measure is a continuous measure as opposed to categorical, allowing a more precise classification of participants’ level of SVO [47]. However, SVO is a narrow personality construct, which may limit the ability to detect individual differences in reputation-based effects. Future studies could also examine if broader personality constructs, such as HEXACO Honesty-Humility or Agreeableness [58] are associated with differential response to reputation-based cues.

### Contributions

This study adds to the literature in several ways. Using established methodology, our aggregate data provide a well-powered attempted replication of the eyes effect (which excludes individual difference data based on SVO). Additionally, our results are suggestive that individual differences may influence how people respond to reputation-based cues. These findings are in the same direction as Simpson and Willer’s (2008; [39]) finding that people who are less prosocial (i.e., SVO egoists) are more likely to calibrate their decisions according to reputation-based cues, whereas SVO prosocials are consistently prosocial. Although our study was underpowered to detect individual differences, our sample size is much larger than the original study [39]. These results can inform future research methodologies; future studies should use observation manipulation with consequences, broader personality variables, and a dependent measure with higher reputational benefits or costs to participants to investigate reputation-based effects.

## Supporting information

S1 Text Supplementary material. (DOCX)

## References

[1] McAuliffe WH, Forster DE, Pedersen EJ, McCullough ME. Experience with anonymous interactions reduces intuitive cooperation. Nature Human Behavior. 2018 Dec;2(12):909. doi: 10.1038/s41562-018-0454-9 30988435

[2] Alexander RD. Foundations of human behavior. The biology of moral systems. Hawthorne, NY, US: Aldine de Gruyter. 1987.

[3] Nowak MA. Five rules for the evolution of cooperation. Science. 2006 Dec 8;314(5805):1560–3. doi: 10.1126/science.1133755 17158317 PMC3279745

[4] Panchanathan K, Boyd R. Indirect reciprocity can stabilize cooperation without the second-order free rider problem. Nature. 2004 Nov;432(7016):499. doi: 10.1038/nature02978 15565153

[5] Rockenbach B, Milinski M. Indirect reciprocity resolves the efficiency dilemma of costly punishment. Nature. 2006;444:718–23. doi: 10.1038/nature05229 17151660

[6] Wedekind C, Milinski M. Cooperation through image scoring in humans. Science. 2000 May 5;288(5467):850–2. doi: 10.1126/science.288.5467.850 10797005

[7] Barclay P, Willer R. Partner choice creates competitive altruism in humans. Proceedings of the Royal Society B: Biological Sciences. 2006 Dec 19;274(1610):749–53. 10.1098/rspb.2006.0209 PMC2197220 17255001

[8] Milinski M, Semmann D, Krambeck H. Donors to charity gain in both indirect reciprocity and political reputation. Proceedings of the Royal Society of London. Series B: Biological Sciences. 2002 May 7;269(1494):881–3. 10.1098/rspb.2002.1964 PMC1690974 12028769

[9] Milinski M, Semmann D, Krambeck HJ. Reputation helps solve the ‘tragedy of the commons’. Nature. 2002 Jan;415(6870):424. doi: 10.1038/415424a 11807552

[10] Smith EA, Bird RL. Turtle hunting and tombstone opening: Public generosity as costly signaling. Evolution and Human Behavior. 2000 Jul 1;21(4):245–61. doi: 10.1016/s1090-5138(00)00031-3 10899477

[11] Capraro V, Giardini F, Vilone D, Paolucci M. Partner selection supported by opaque reputation promotes cooperative behavior. Judgment and Decision Making. 2016 Nov 6;11(6):589–600.

[12] Andreoni J, Petrie R. Public goods experiments without confidentiality: a glimpse into fund-raising. Journal of Public Economics. 2004 Jul 1;88(7–8):1605–23.

[13] Barclay P. Trustworthiness and competitive altruism can also solve the “tragedy of the commons”. Evolution and Human Behavior. 2004 Jul 1;25(4):209–20.

[14] Bradley A, Lawrence C, Ferguson E. Does observability affect prosociality?. Proceedings of the Royal Society B: Biological Sciences. 2018 Mar 28;285(1875):20180116. doi: 10.1098/rspb.2018.0116 29593114 PMC5897647

[15] Rege M, Telle K. The impact of social approval and framing on cooperation in public good situations. Journal of Public Economics. 2004 Jul 1;88(7–8):1625–44.

[16] Sylwester K, Roberts G. Cooperators benefit through reputation-based partner choice in economic games. Biology Letters. 2010 Apr 21;6(5):659–62. doi: 10.1098/rsbl.2010.0209 20410026 PMC2936156

[17] Piazza J, Bering JM. Concerns about reputation via gossip promote generous allocations in an economic game. Evolution and Human Behavior. 2008 May 1;29(3):172–8.

[18] Bateson M, Nettle D, Roberts G. Cues of being watched enhance cooperation in a real-world setting. Biology Letters. 2006 Jun 27;2(3):412–4. doi: 10.1098/rsbl.2006.0509 17148417 PMC1686213

[19] Burnham TC, Hare B. Engineering human cooperation. Human Nature. 2007 Jun 1;18(2):88–108. doi: 10.1007/s12110-007-9012-2 26181843

[20] Haley KJ, Fessler DM. Nobody’s watching?: Subtle cues affect generosity in an anonymous economic game. Evolution and Human Behavior. 2005 May 1;26(3):245–56.

[21] Kawamura Y, Kusumi T. The norm-dependent effect of watching eyes on donation. Evolution and Human Behavior. 2017 Sep 1;38(5):659–66.

[22] Nettle D, Harper Z, Kidson A, Stone R, Penton-Voak IS, Bateson M. The watching eyes effect in the Dictator Game: it’s not how much you give, it’s being seen to give something. Evolution and Human Behavior. 2013 Jan 1;34(1):35–40.

[23] Sparks A, Barclay P. Eye images increase generosity, but not for long: The limited effect of a false cue. Evolution and Human Behavior. 2013 Sep 1;34(5):317–22.

[24] Sparks A, Barclay P. No effect on condemnation of short or long exposure to eye images. Letters on Evolutionary Behavioral Science. 2015 Aug 17;6(2):13–6.

[25] Ernest-Jones M, Nettle D, Bateson M. Effects of eye images on everyday cooperative behavior: a field experiment. Evolution and Human Behavior. 2011 May 1;32(3):172–8.

[26] Francey D, Bergmüller R. Images of eyes enhance investments in a real-life public good. PLoS One. 2012 May 18;7(5):e37397. doi: 10.1371/journal.pone.0037397 22624026 PMC3356250

[27] Powell KL, Roberts G, Nettle D. Eye images increase charitable donations: Evidence from an opportunistic field experiment in a supermarket. Ethology. 2012 Nov;118(11):1096–101.

[28] Mifune N, Hashimoto H, Yamagishi T. Altruism toward in-group members as a reputation mechanism. Evolution and Human Behavior. 2010 Mar 1;31(2):109–17.

[29] Oda R, Niwa Y, Honma A, Hiraishi K. An eye-like painting enhances the expectation of a good reputation. Evolution and Human Behavior. 2011 May 1;32(3):166–71.

[30] Rigdon M, Ishii K, Watabe M, Kitayama S. Minimal social cues in the dictator game. Journal of Economic Psychology. 2009 Jun 1;30(3):358–67.

[31] BourratP, BaumardN, McKayR. Surveillance cues enhance moral condemnation. Evolutionary Psychology. 2011 Apr 1;9(2):147470491100900206.22947966

[32] Dear K, Dutton K, Fox E. Do ‘watching eyes’ influence antisocial behavior? A systematic review & meta-analysis. Evolution and Human Behavior. 2019 May 1;40(3):269–80.

[33] Brudermann T, Bartel G, Fenzl T, Seebauer S. Eyes on social norms: A field study on an honor system for newspaper sale. Theory and decision. 2015 Sep 1;79(2):285–306.

[34] Cai W, Huang X, Wu S, Kou Y. Dishonest behavior is not affected by an image of watching eyes. Evolution and Human Behavior. 2015 Mar 1;36(2):110–6.

[35] Lamba S, Mace R. People recognise when they are really anonymous in an economic game. Evolution and Human Behavior. 2010 Jul 1;31(4):271–8.

[36] Matsugasaki K, Tsukamoto W, Ohtsubo Y. Two failed replications of the watching eyes effect. Letters on Evolutionary Behavioral Science. 2015 Sep 9;6(2):17–20.

[37] Raihani NJ, Bshary R. A positive effect of flowers rather than eye images in a large-scale, cross-cultural dictator game. Proceedings of the Royal Society B: Biological Sciences. 2012 Jun 6;279(1742):3556–64. doi: 10.1098/rspb.2012.0758 22673357 PMC3396908

[38] Northover SB, Pedersen WC, Cohen AB, Andrews PW. Artificial surveillance cues do not increase generosity: Two meta-analyses. Evolution and Human Behavior. 2017 Jan 1;38(1):144–53.

[39] Simpson B, Willer R. Altruism and indirect reciprocity: The interaction of person and situation in prosocial behavior. Social Psychology Quarterly. 2008 Mar;71(1):37–52.

[40] Declerck CH, Boone C, Kiyonari T. No place to hide: When shame causes proselfs to cooperate. The Journal of Social Psychology. 2014 Jan 2;154(1):74–88. doi: 10.1080/00224545.2013.855158 24689338

[41] Baumert A, Schlösser T, Schmitt M. Economic Games. European Journal of Psychological Assessment. 2014.

[42] Galizzi MM, Navarro-Martínez D. On the external validity of social preference games: a systematic lab-field study. Management Science. 2018 Feb 13;65(3):976–1002.

[43] Hilbig BE, Glöckner A, Zettler I. Personality and prosocial behavior: Linking basic traits and social value orientations. Journal of Personality and Social Psychology. 2014 Sep;107(3):529. doi: 10.1037/a0036074 25019254

[44] Peysakhovich A, Nowak MA, Rand DG. Humans display a ‘cooperative phenotype’that is domain general and temporally stable. Nature Communications. 2014 Sep 16;5:4939. doi: 10.1038/ncomms5939 25225950

[45] Wu J, Balliet D, Tybur JM, Arai S, Van Lange PA, Yamagishi T. Life history strategy and human cooperation in economic games. Evolution and Human Behavior. 2017 Jul 1;38(4):496–505.

[46] Van Lange PA. The pursuit of joint outcomes and equality in outcomes: An integrative model of social value orientation. Journal of Personality and Social Psychology. 1999 Aug;77(2):337.

[47] Murphy RO, Ackermann KA, Handgraaf M. Measuring social value orientation. Judgment and Decision Making. 2011 Dec;6(8):771–81.

[48] Christie R, Geis FL. Studies in machiavellianism. Academic Press; 2013 Oct 22.

[49] Team RC. R: A language and environment for statistical computing.

[50] Cumming G. Understanding the new statistics: effect sizes. Confidence Intervals, and Meta-Analysis. 2012.

[51] Phillips ND. Yarrr! The pirate’s guide to R. APS Observer. 2017 Feb 28;30(3).

[52] Dowle M, Srinivasan A, Gorecki J, Chirico M, Stetsenko P, Short T, et al. Package ‘data. table’. Extension of ‘data. frame. 2019 Dec 9.

[53] Lüdecke D. sjstats: Statistical functions for regression models. R package version 0.14. 2018;3.

[54] Stanley D. ApaTables: Create american psychological association (apa) style tables. R package version. 2018;1(1).

[55] Navarro D. lsr: Companion to “Learning Statistics with R”. R package version 0.5. 2015.

[56] Revelle WR. psych: Procedures for personality and psychological research. 2017.

[57] Grosch K, Rau HA. Gender differences in honesty: The role of social value orientation. Journal of Economic Psychology. 2017 Oct 1;62:258–67.

[58] Brañas-Garza P, Capraro V, Rascon-Ramirez E. Gender differences in altruism on Mechanical Turk: Expectations and actual behaviour. Economics Letters. 2018 Sep 1;170:19–23.

[59] Balliet D, Li NP, Macfarlan SJ, Van Vugt M. Sex differences in cooperation: a meta-analytic review of social dilemmas. Psychological Bulletin. 2011 Nov;137(6):881. doi: 10.1037/a0025354 21910518

[60] Ashton MC, Lee K. Empirical, theoretical, and practical advantages of the HEXACO model of personality structure. Personality and Social Psychology Review. 2007 May;11(2):150–66. doi: 10.1177/1088868306294907 18453460

