# Supplementary material for: No effect of ‘watching eyes’: An attempted replication and extension investigating individual differences
Source: PLoS One. 2021 Oct 6;16(10):e0255531. doi: 10.1371/journal.pone.0255531 (PMC8494318; doi:10.1371/journal.pone.0255531)
Supplement: S1 Text — (DOCX) [file pone.0255531.s001.docx]

**Supplementary Material**

**Table of Contents**

| Social Value Orientation Distributions, by population | | ...………………………………2 |
| --- | --- | --- |
| Participant generated ID | ……………………………………………………………2 | |
| Exploratory Scales Included | ……………………………………………………………3 | |
| Eyes Program Description | ……………………………………………………………4 | |
| Supplemental Results | ……………….……….…………………………………12 | |

**Social Value Orientation Distribution by Population**

**Table S1.**

**Classifications of Social Value Orientations (SVO) from the Triple-Dominance Measure (Van Lange, 1999)**

| Sample | Sample Characteristics | Total *N* | *n* Prosocials (%) | *n*  Egoists (%) | *n* Competitors (%) | Unclassifi-able |
| --- | --- | --- | --- | --- | --- | --- |
| University of Guelph 2016^1^ | First-year Psychology Undergrads | 1792 | 1113 (62%) | 314  (18%) | 181  (10%) | 184  (10%) |
| University of Guelph 2017^1^ | First-year Psychology Undergrads | 1170 | 648  (56%) | 261  (22%) | 97  (8%) | 164  (14%) |
| University of Regina^1^ | Business Undergrads  (all levels) | 230 | 93  (40%) | 61  (27%) | 26  (11%) | 50  (22%) |
| Amazon Mechanical Turk^2^ | US Crowdsourced Sample | 2213 | 1459  (66%) | 682  (31%) | 72  (3%) | - |

^1^*Note.* Data from pre-experimental survey, not only participants invited to participate in the study.

^2^*Note.* This was part of a recruitment for a different project, and is based on the responses of a single SVO item rather than the whole scale (Rotella & Barclay, 2020).

**Participant-Generated ID**

To anonymously link data from the pre-term survey to the data collected in lab, we had participants self-generate their own ID. Participants were prompted with the following questions to create a unique code:

1. First letter of the month you were born
2. Third letter of your first name
3. Second letter of your last name
4. Second letter of the street you live on
5. Third letter of the town you were born
6. The number of siblings you have as of September 1^st^ of this year

*Note*: These questions varied slightly, depending on the term of data collection.

**Exploratory Scales**

*Machiavellianism (Mach-IV)*. To measure Machiavellianism, we used the Mach-IV inventory (Christie & Geis, 1970). This consisted of 20 items, which were evaluated using a 5-point Likert scale. Some example items are “Anyone who completely trusts anyone else is asking for trouble” and “It is possible to be good in all respects”. After reverse coding the appropriate items, scores were aggregated so that higher scores indicated greater Machiavellianism.

*SVO Slider Measure.* The SVO Slider Measure (Murphy, Ackermann, & Handgraaf, 2011) is a continuous measure of cooperative preferences, assessed using six items. Participants chose their preferred distribution of points, which would be allocated between themselves and a hypothetical other person (e.g., “100 points for you, 50 points for other”). Choices were aggregated and converted to a number on a Cartesian plane, where higher values are associated greater prosociality.

**Program Description – Eyes Condition**

1. Experimenter enters participant ID and presses ‘Next Page’:


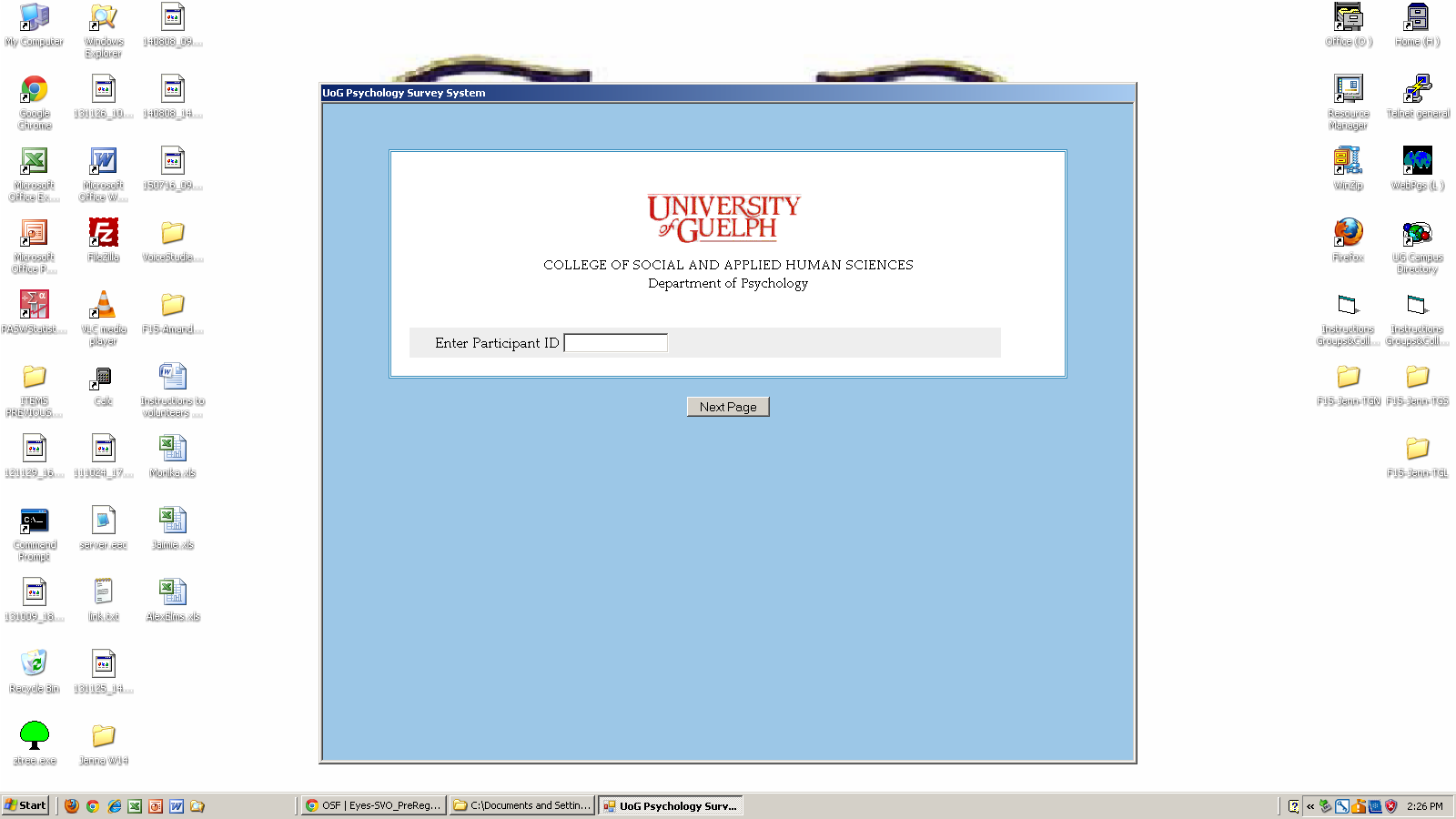


2. When participants sit down, they view the demographics survey:


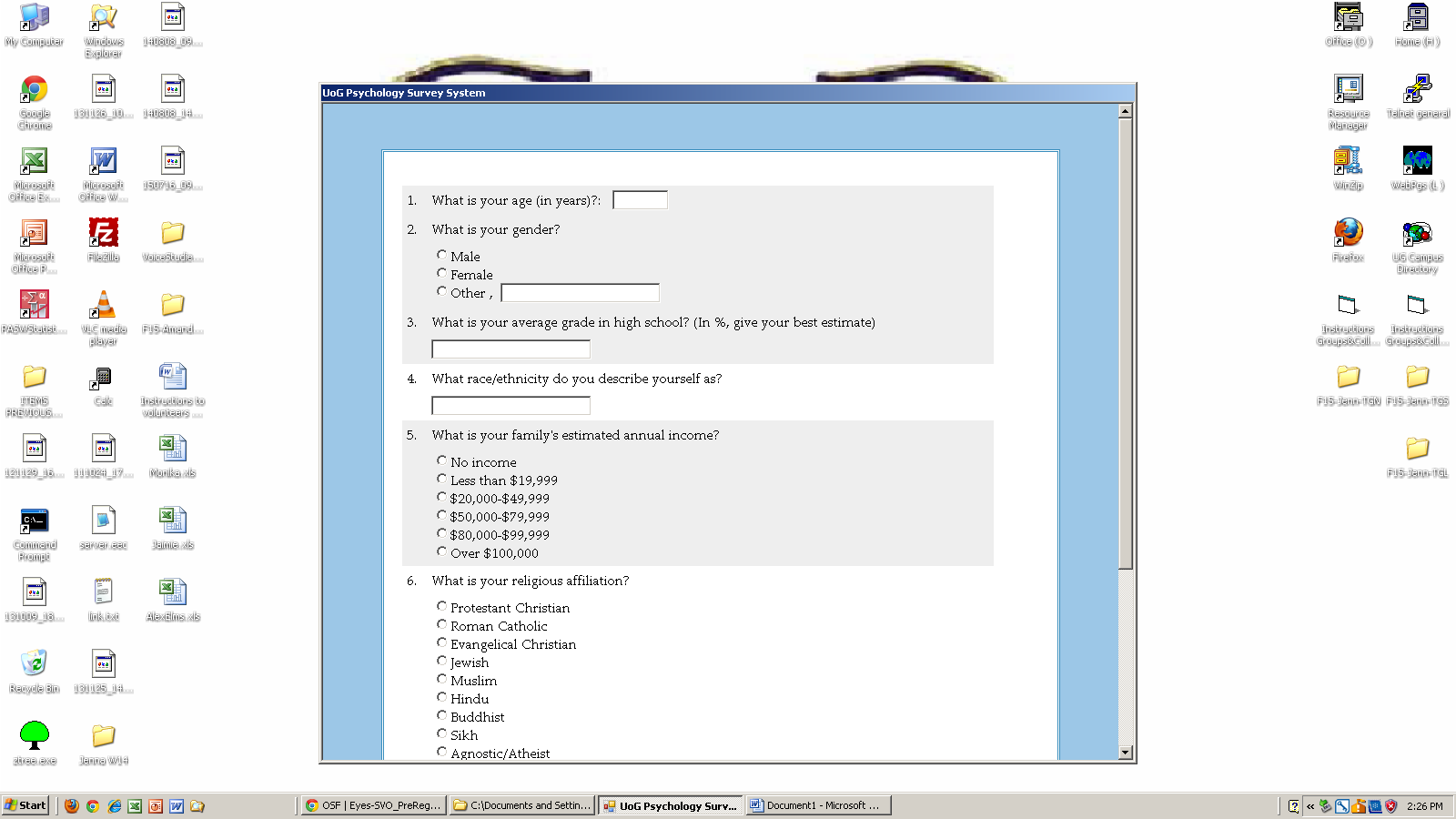


3. When participants are finished demographics questions, they read game instructions:


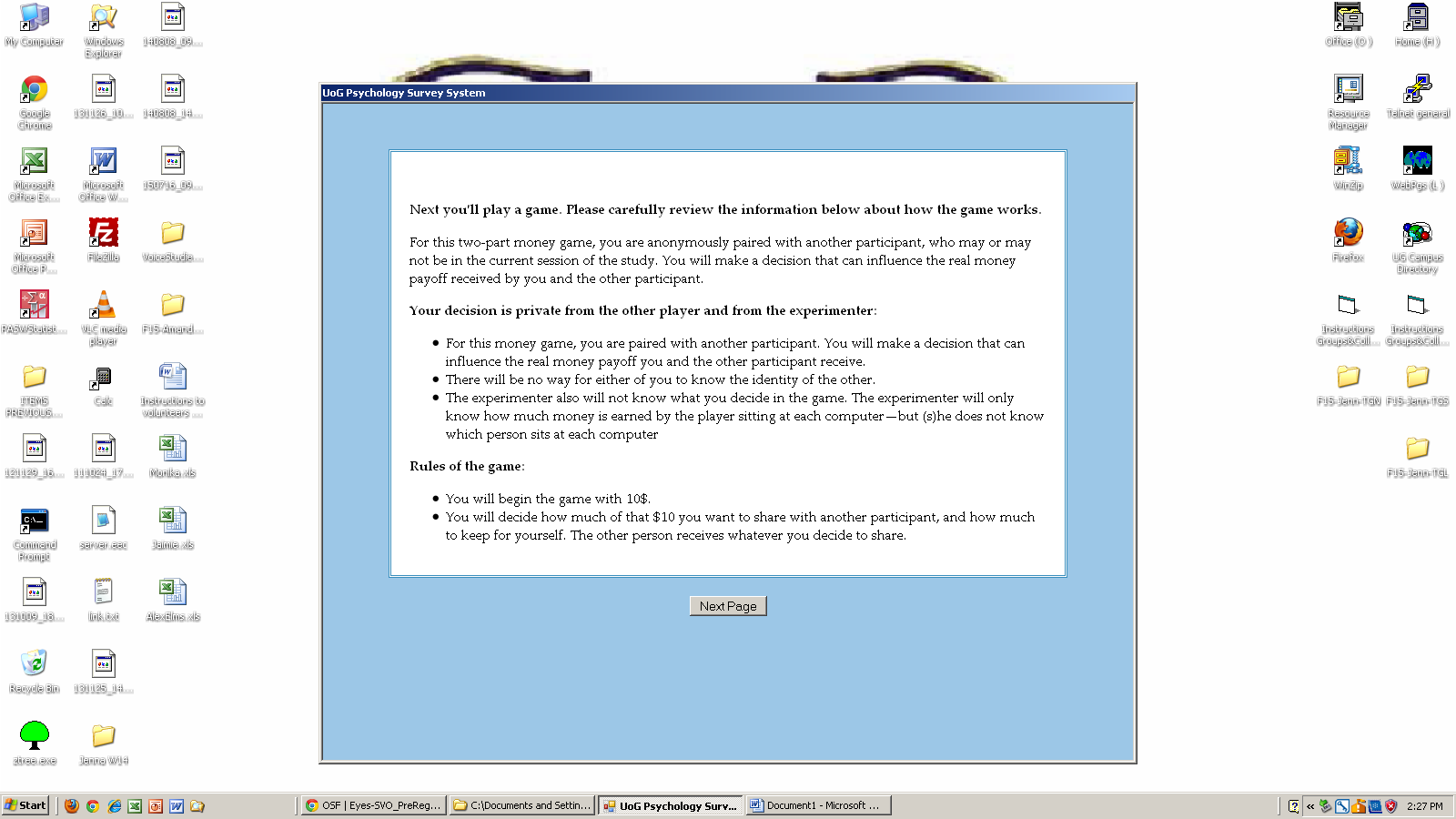


4. Program instructs participants that it is loading the game:


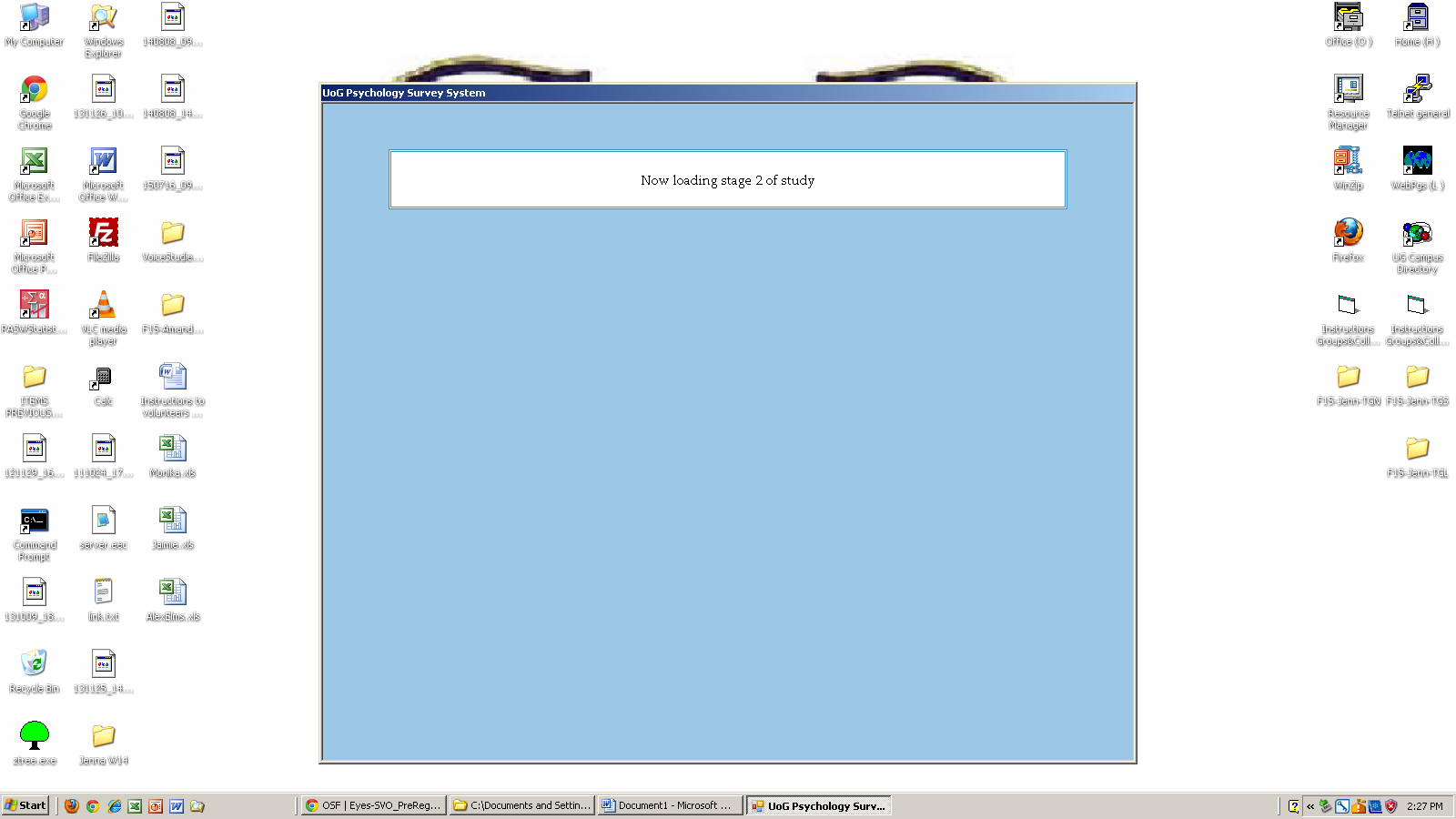


5. Window minimizes


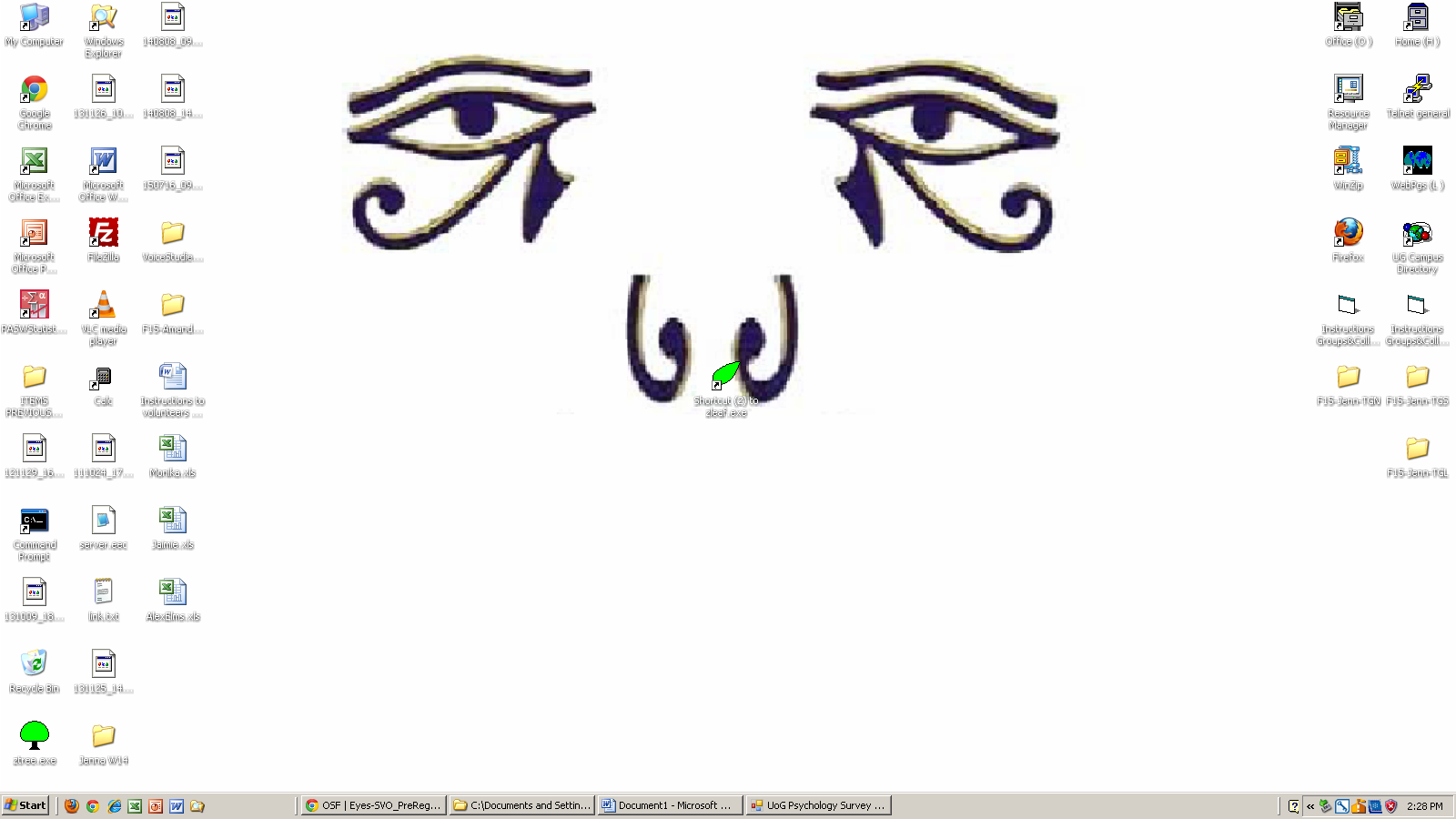


6. Re-sized window loads, participants play dictator game


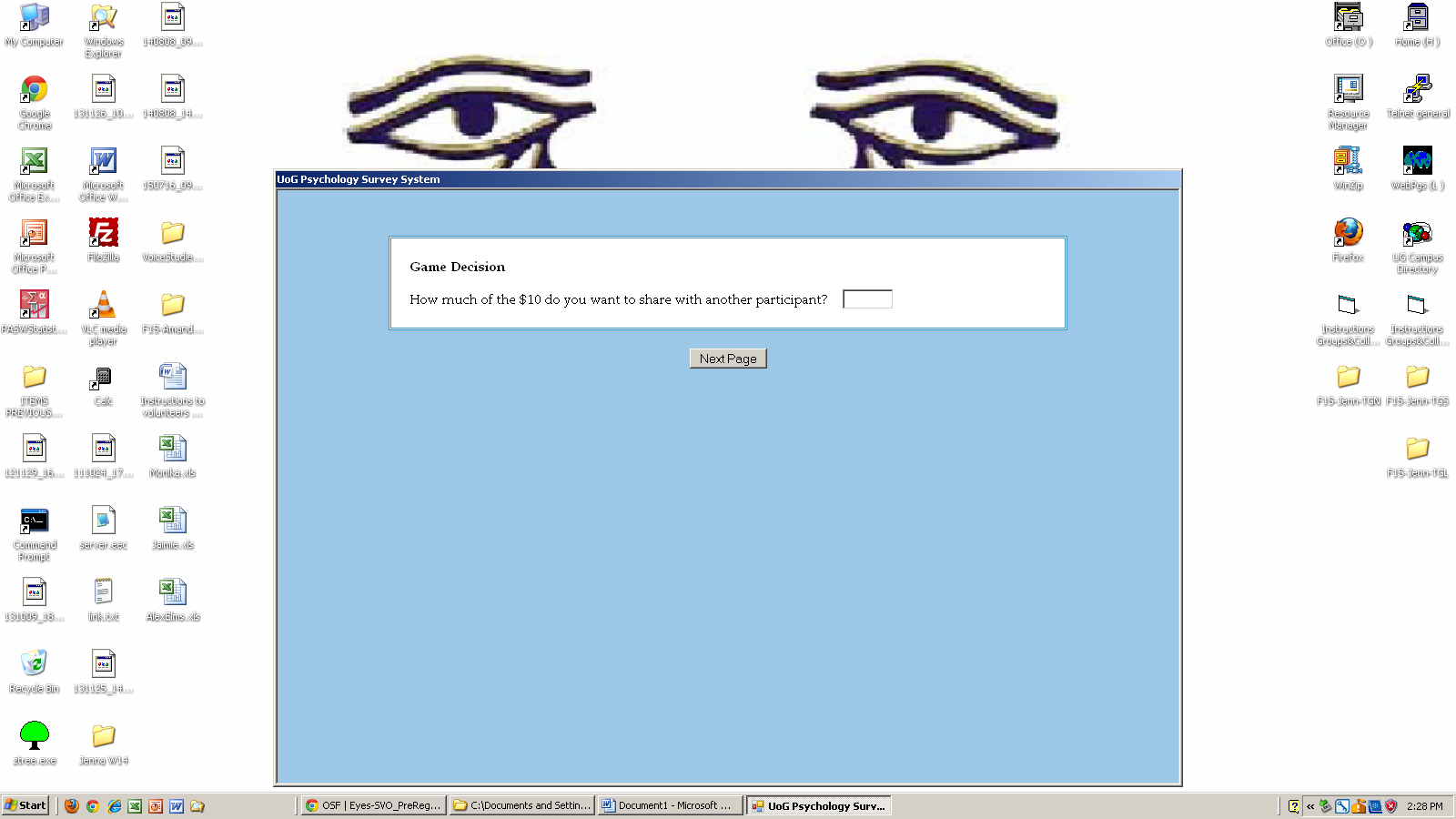


7. Program thanks participants for completing the program


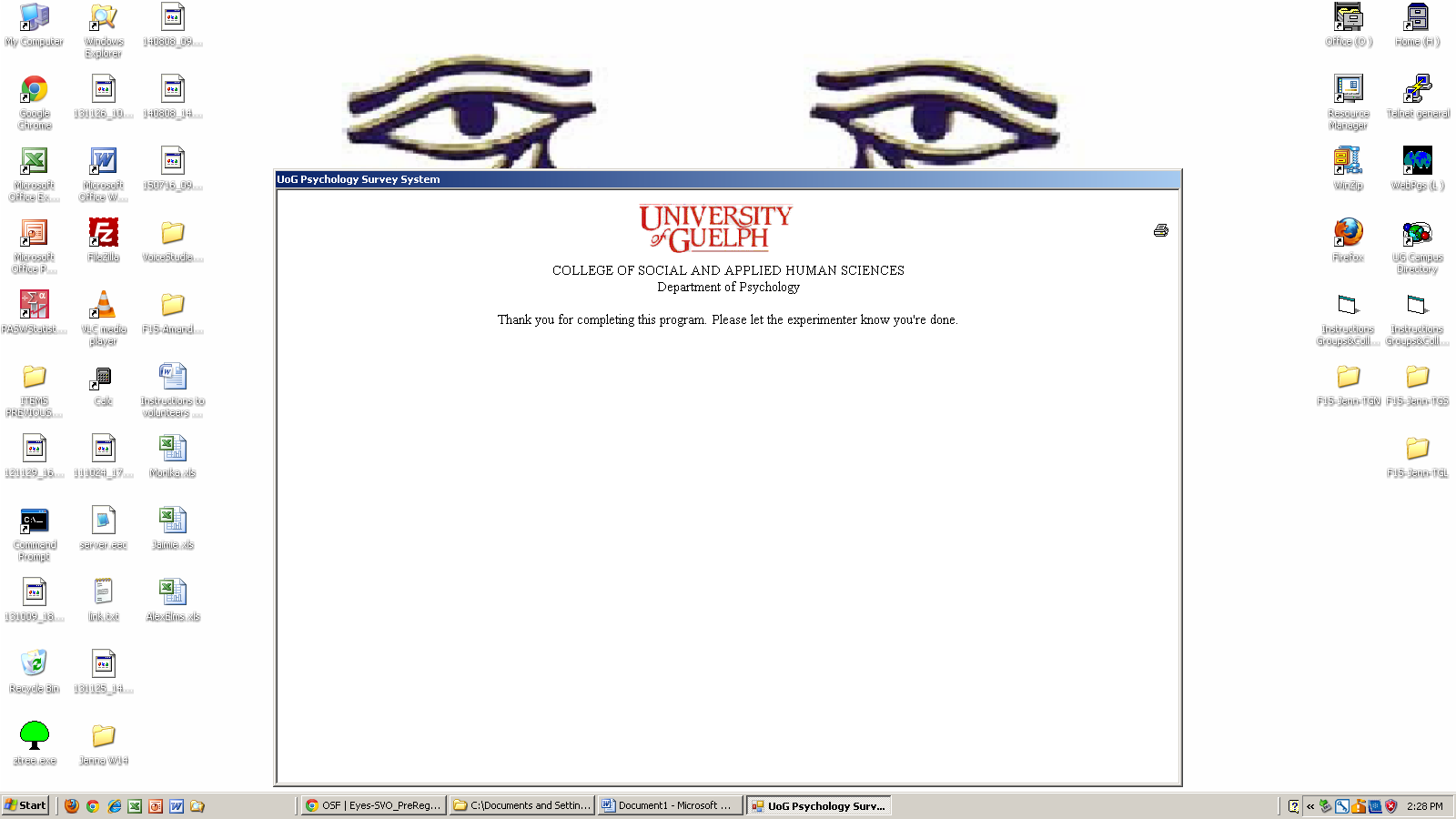


**Program Description – No Eyes and Public conditions**

1. Experimenter enters participant ID and presses ‘Next Page’:


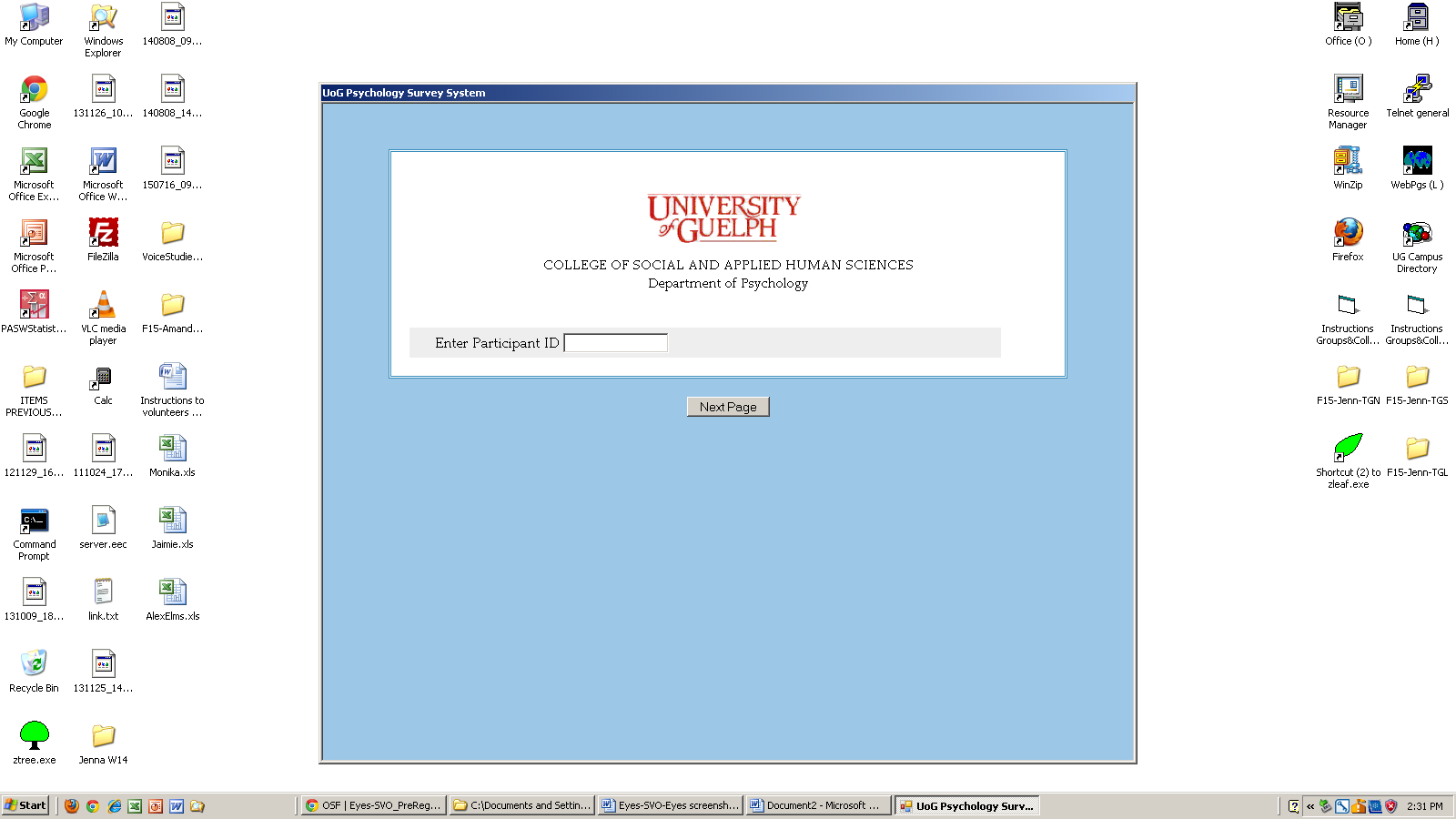


2. When participants sit down, they view the demographics survey:


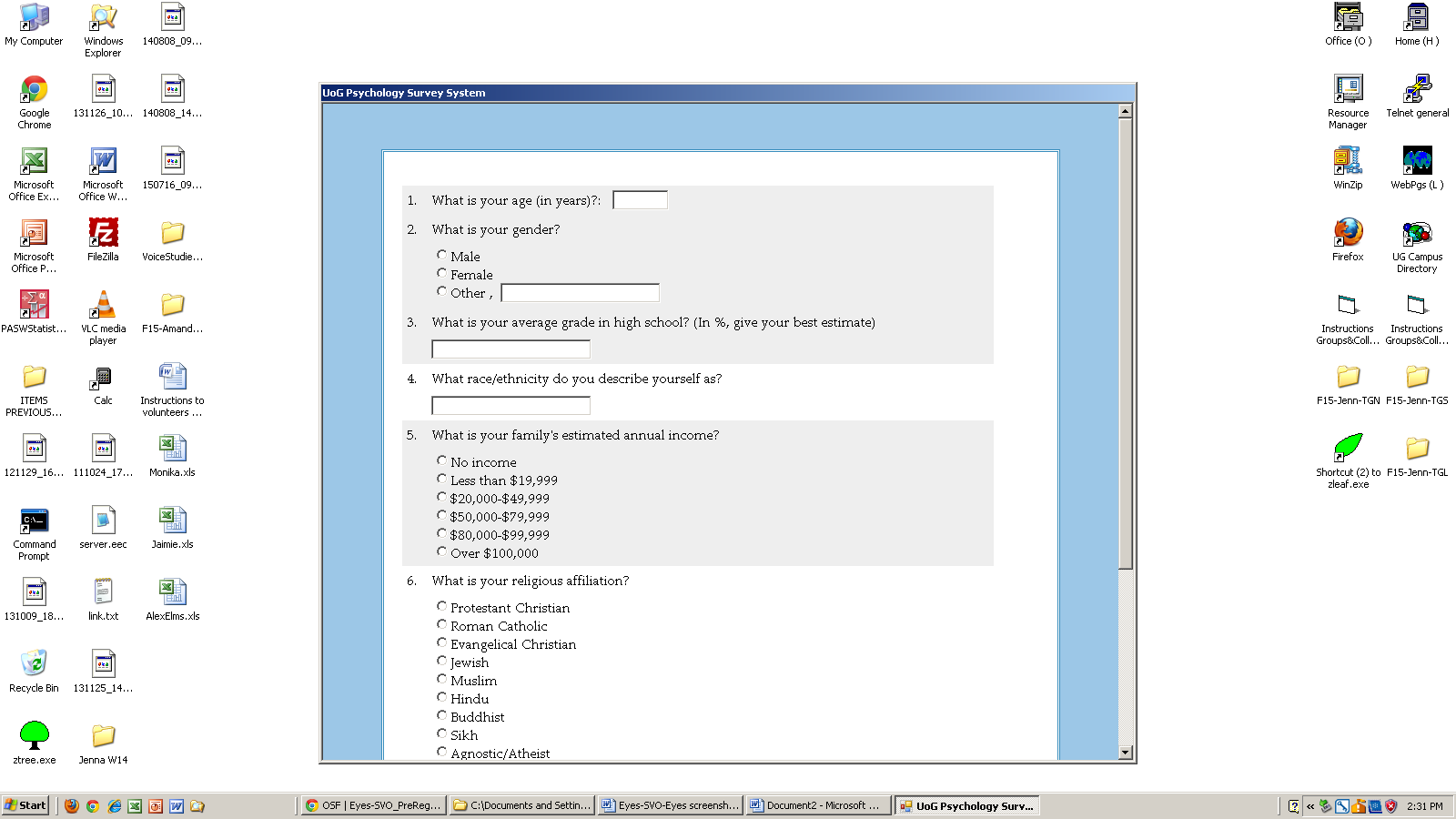


3. When participants are finished demographics questions, they read game instructions:


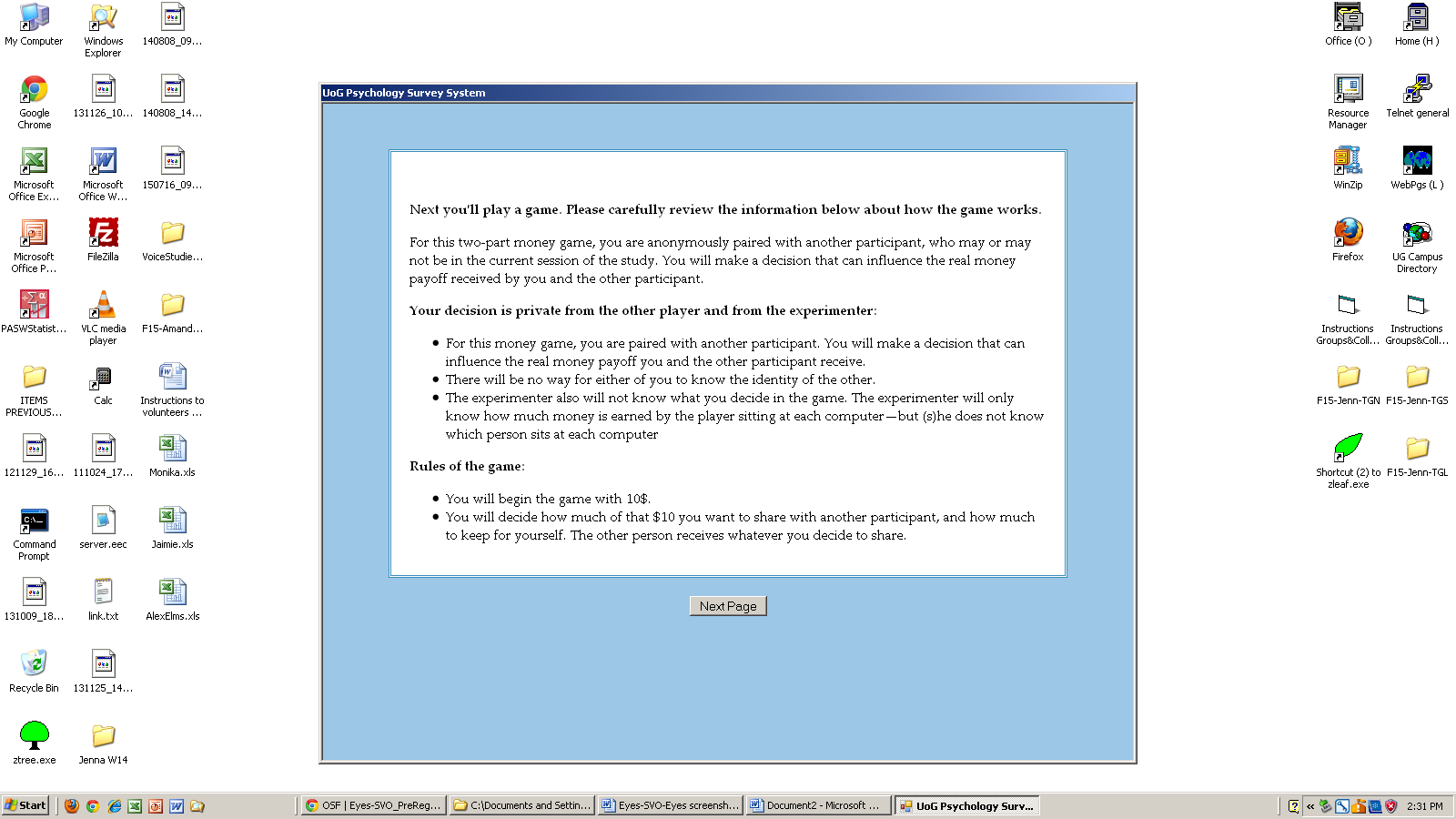


4. Program instructs participants that it is loading the game:


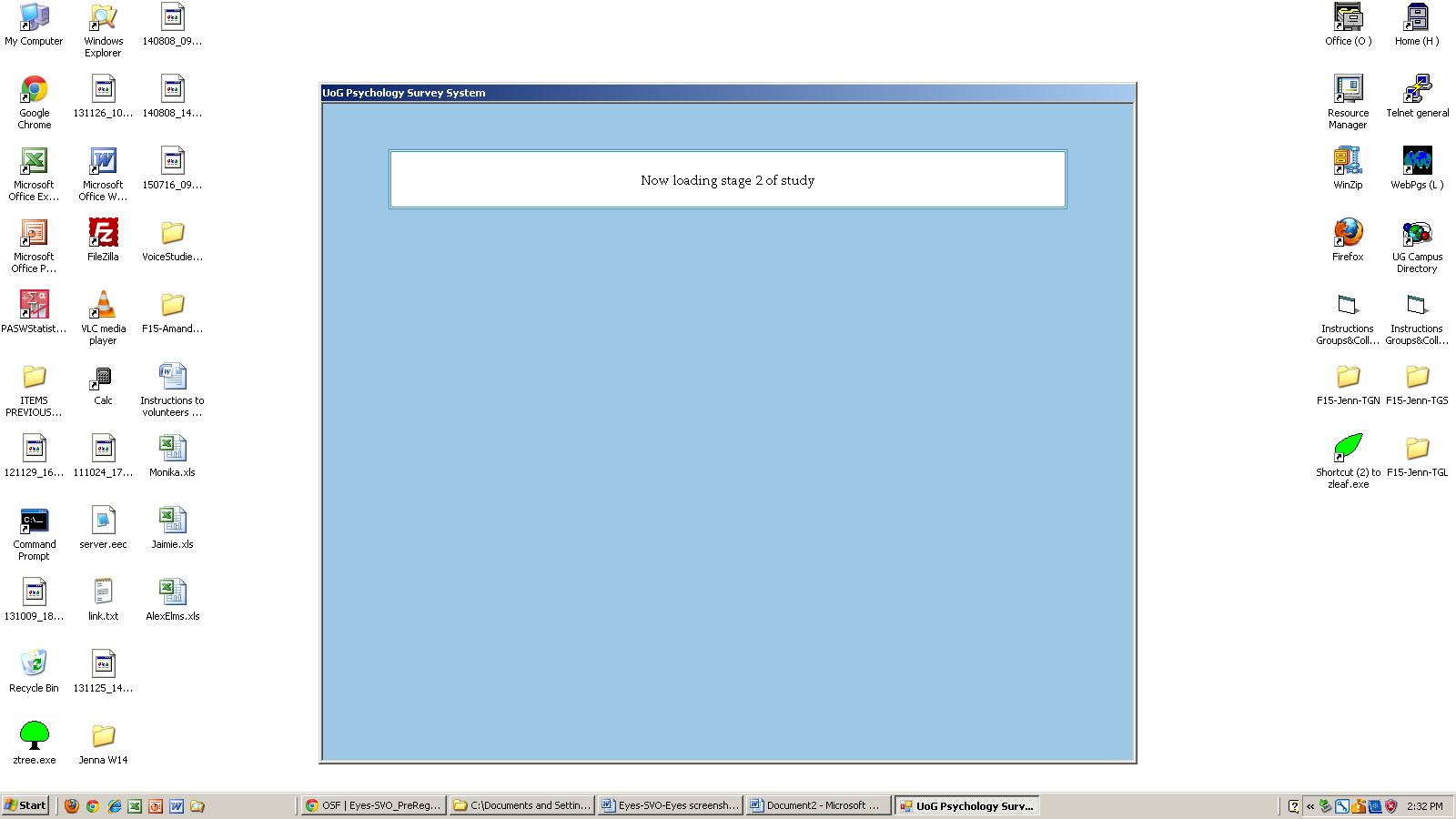


5. Window minimizes


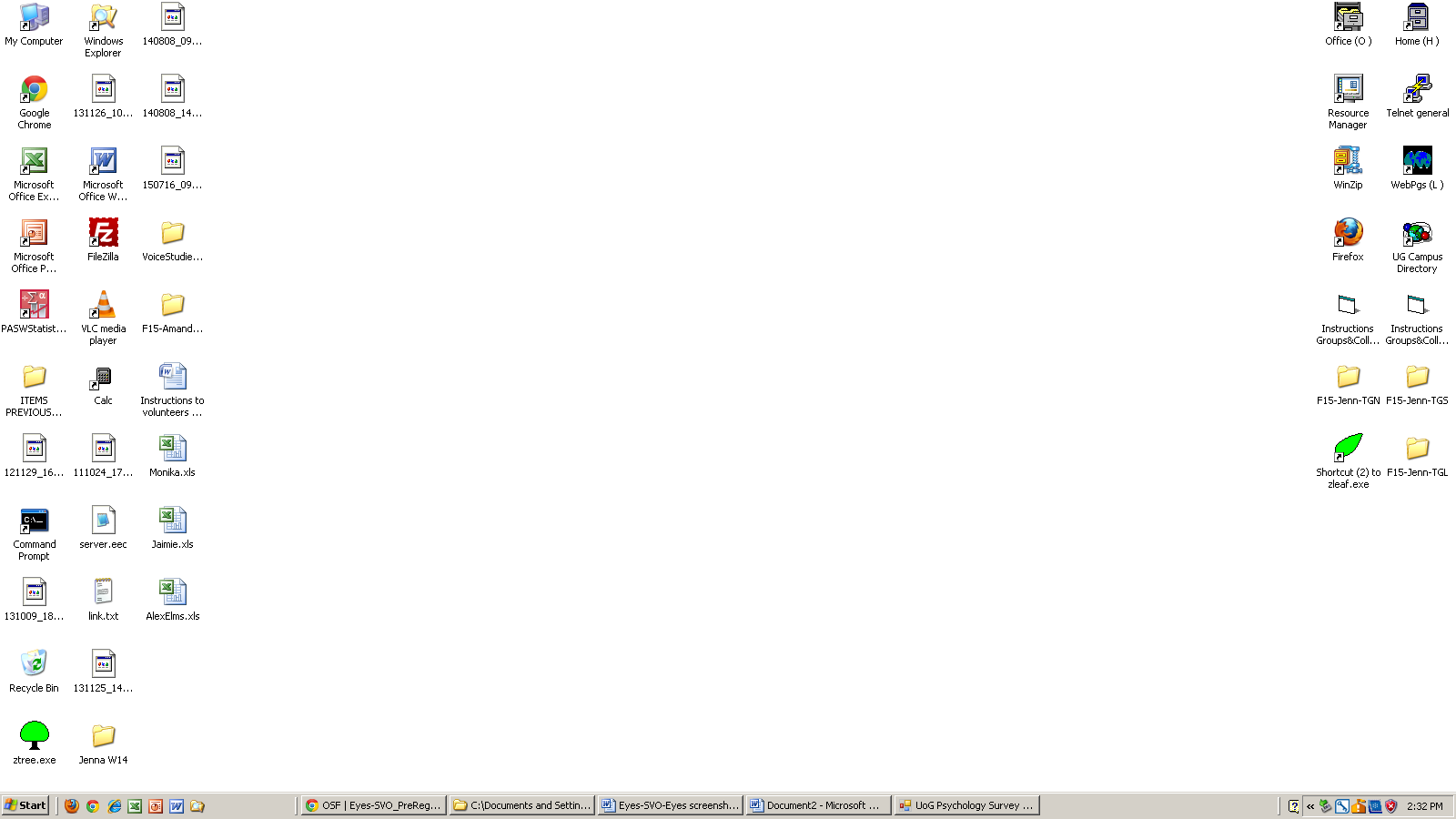


6. Re-sized window loads, participants play dictator game


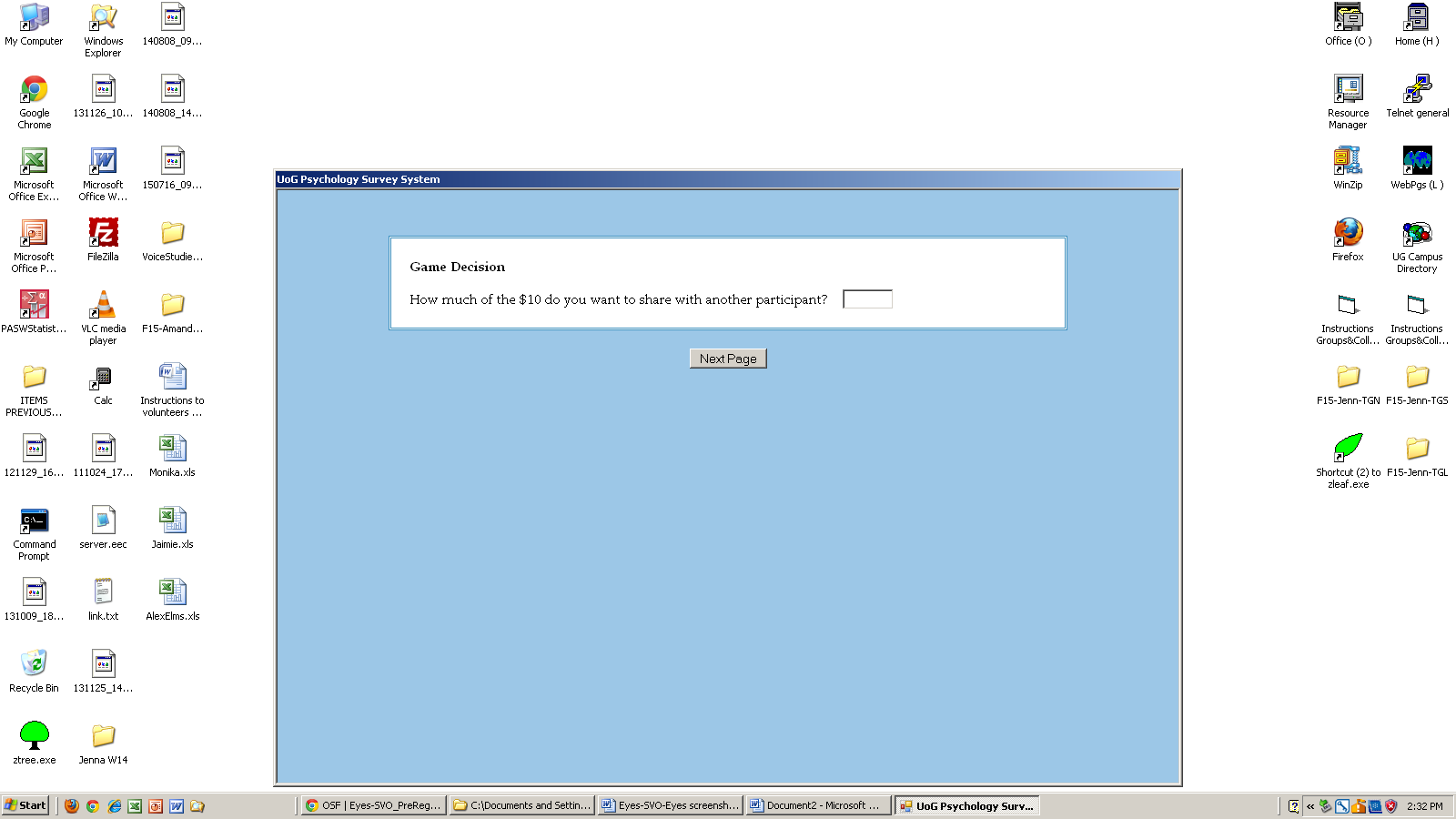


7. Program thanks participants for completing the program


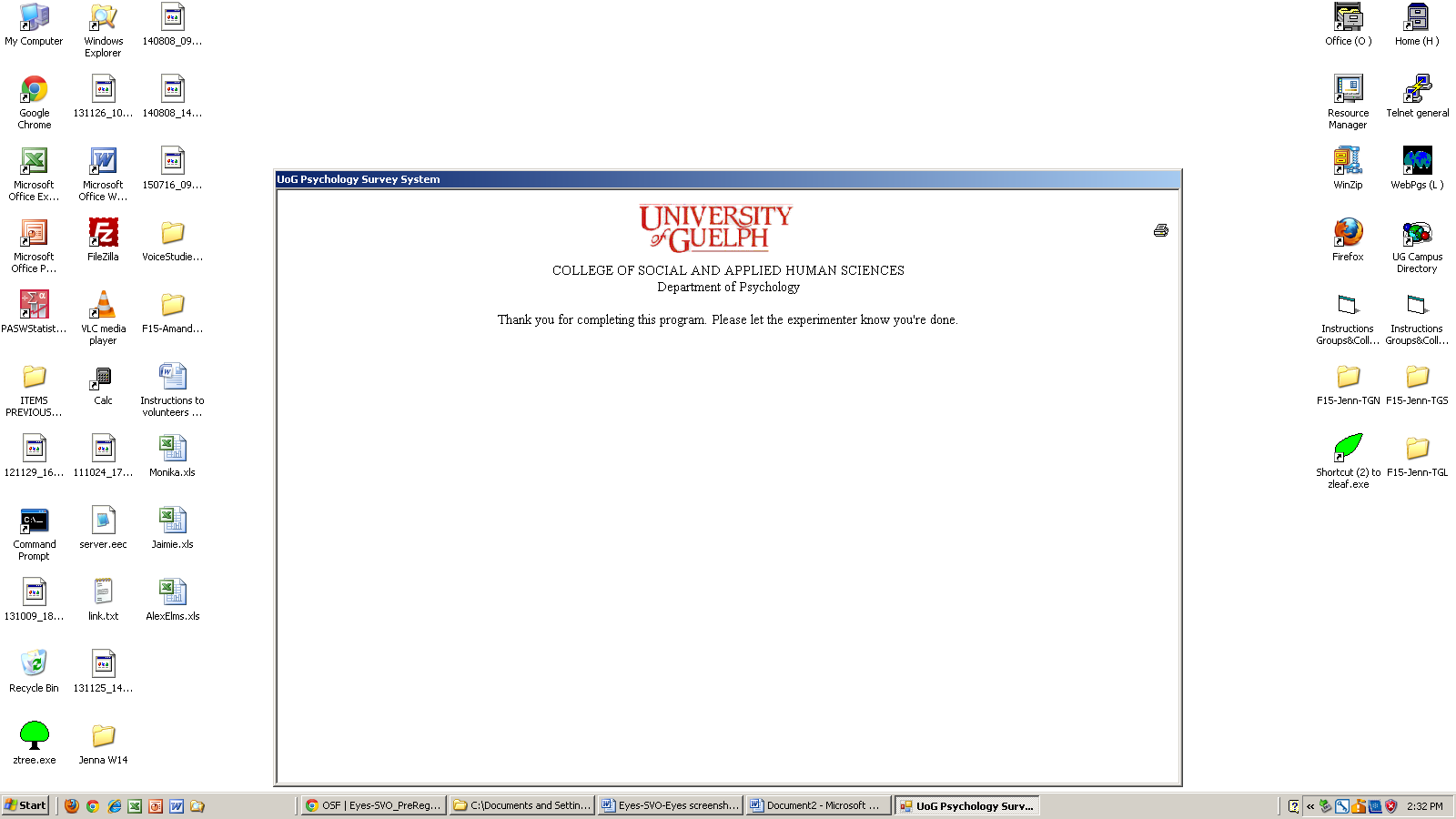


**Supplemental Results**

**Perceptions of Observation and Dictator Game Allocations**

Perceptions of observation were not related to dictator game allocations, across any of the conditions. See Table S2.

**Table S2**

|  | Question | | |
| --- | --- | --- | --- |
| Condition | “*Someone is watching the amount of money I allocate to the recipient*.” | “*The experimenter will see the amount of money I allocated and form a poor opinion of me*” | “*A situation in which other people would find out how I behaved*” AND “*A situation in which other people would see my behavior*” |
| All data | *r* = .08, 95CI[-.03, .18]  *p* = .139 | *r* = -.09, 95CI[-.19, .02]  *p* = .096 | *r* = .03, 95CI[-.07, .13]  *p* = .583 |
| Public | *r* = .17, 95CI[-.03, .35]  *p* = .095 | *r* = .17, 95CI[-.23, .17]  *p* = .763 | *r* = .11, 95CI[-.09, .30]  *p* = .283 |
| Eyes | *r* = .07, 95CI[-.11, .26]  *p* = .438 | *r* = -.03, 95CI[-.22, .16]  *p* = .738 | *r* = .05, 95CI[-.14, .23]  *p* = .625 |
| No Eyes | *r* = -.05, 95CI[-.24, .14]  *p* = .593 | *r* = -.19, 95CI[-.37, -.01]  *p* = .044 | *r* = -.11, 95CI[-.29, .07]  *p* = .236 |

**Post-Experimental Questionnaire**

To determine which factors influenced decisions in the dictator game, and how they varied by condition, we analyzed the responses on the post-experimental questionnaire. These analyses are based on the ones presented in Oda, Niwa, Honma, & Hirashi (2011). Note, the following analyses were conducted in SPSS 26.

First, we began by correlating the 17-items on the post-experimental questionnaire, which were found to be correlate with one another (*r* = -.21 to .74). Thus, to analyze this data we conducted a principal component analysis. Three principle components were extracted with Eigen values greater than 1, which explained 60.39% of variance. *Table S3.2* contains the factor loadings for each component, and the component score matrices.

We used a regression analysis to determine whether the components predicted the amount of money allocated to recipients in the dictator game. All three components predicted dictator game allocations, *F*(3,352) = 65.25, *p* < .001, *R* = .60, *R^2^* = .36, as presented in *Table S3.2*.

We then used a MANOVA to determine the component differed by experimental condition (see Table S3.3). Only the first component differed by condition, *F*(2,330) = 12.62, *p* < .001, *η_p_^2^* *=* .072; the other two components did not vary by condition, *F*(2,330) = 0.98, *p* = .376, and *F*(2,330) = 0.542 *p* = .592, respectively. Simple main effects with Tukey’s HSD indicated that there were higher scores for Component 1 in the Public condition compared to the Eyes condition (*M_diff_* = -0.62, *p* < .001, 95CI[-0.92, -0.31]) and the No Eyes condition, *M_diff_* = -0.51, *p* < .001, 95CI[-0.81, -0.20. There were no differences between the Eyes and No Eyes conditions, *M_diff_* = -0.11, *p* =.652, 95CI[-0.41, 0.18]. See *Figure S3.2*. These analyses support the information in text, where participants perceived to be seen more in the Public condition than the other conditions, and that the Eyes and No Eyes conditions were similarly perceived.

Lastly, we used a MANOVA to determine if responses on the post-experimental questionnaire differed by SVO. The second component of the PCA differed by SVO, *F*(2, 366) = 5.33, *p* = .005, *η_p_^2^* *=* .026, with prosocials (*M* = 0.04, *SD* = 0.92) having higher scores than egoists (*M* = -0.34, *SD* = 1.08). The first and third components of the PCA did not vary by SVO, *F*(2, 366) = 1.80, *p* = .165, *η_p_^2^* *=* .010, and *F*(2, 366) = 0.07, *p* = .936, *η_p_^2^* *=* .000, respectively. This finding is not surprising, given that the items that loaded onto this component include “I only have to give as much money as I want to regardless of the recipient's desire”, “I should think of the recipient”, and “I will feel guilty if I don't share an equal amount with the recipient”, suggesting that this component is related to concern for the recipient, a prosocial sentiment.

## Tests of Equivalence (TOST)

Does failure to replicate the eyes effect show evidence that there is indeed no effect? We cannot make this conclusion with standard null hypothesis significance testing, because a non-significant result does not prove the null hypothesis. A non-significant result means that we cannot *reject* an effect size of zero but does not mean that we should *accept* an effect size of zero. To see whether our results are consistent with zero effect size, we use tests of equivalence (e.g., Lakens, 2017; Lakens, Scheel, & Isager, 2018). Tests of equivalence allow researchers to say that their effects are significantly smaller than some minimum level of interest (e.g., a minimum effect size), such that the effect is small enough to be equivalent to zero for practical purposes.

To see whether our effect sizes are smaller than the smallest effect size of interest, we used the TOST procedure which uses two one-sided t-tests with a specified equivalence bound.

**Main effect of eyes**

For the main effect of eyes, we set the minimum effect size of interest to be Cohen’s *d* of 0.42, which was the effect size of interest we attempted to replicate (see Lakens, Scheel, & Isager, 2018). Because a *d* of 0.42 is considered a medium effect size, we replicated these analyses with a small effect (*d* = 0.20) and contrast results below.

***Eyes vs No Eyes.*** The effect observed is statistically equivalent, because the larger of the two *p* values is less than .05, *t*(240.49) = 3.03, *p* = .001. This indicates that we can reject the hypothesis that the true effect is smaller than *d* = -0.42 or larger than *d* = 0.42 (see Figure S4). In other words, if an effect exists the effect size is smaller than in previous studies, and the Eyes and No Eyes conditions are statistically equivalent in this experiment. The effect became statistically equivalent at a Cohen’s *d* of 0.24, suggesting a small effect.

When we replicated the TOST procedure using Cohen’s *d* of 0.20, we found that the observed effect was not statistically equivalent, *t*(240.49) = 1.30, *p* = .098. This is consistent with our interpretation above, which suggests that the effect size is smaller than in previous studies, but larger than *d* = 0.20. The present study was underpowered to find an effect of this size.

***Public vs No Eyes.*** Again, the effect observed when comparing the Public condition to the No Eyes condition is statistically equivalent, because the larger of the two *p* values is less than .05, *t*(224.43) = -2.09, *p* = .019. Thus, we reject the hypothesis that the true effect is smaller than *d* = -0.42 or larger than *d* = 0.42. In this experiment, the Public and No Eyes conditions are statistically equivalent, and if an effect exists the effect size is smaller than anticipated.

When we replicated the TOST procedure using Cohen’s *d* of 0.20, we found that the observed effect was not statistically equivalent, *t*(224.43) = -0.41, *p* = .340. This suggests that the effect size is smaller than in previous studies, but larger than *d* = 0.20. Again, this study was underpowered to find an effect of this size. The effect became statistically equivalent at a Cohen’s *d* of 0.37, suggesting a small-medium effect between the public and control conditions.

***Public vs Eyes.*** Lastly, the effect between the Public condition to the Eyes condition was statistically equivalent, *t*(229.74) = 1.90, *p* = .029. Thus, we reject the hypothesis that the true effect is smaller than *d* = -0.42 or larger than *d* = 0.42.

When we replicated the TOST procedure using Cohen’s *d* of 0.20, we found that the observed effect was not statistically equivalent, *t*(229.74) = 0.22, *p* = .411. This is consistent with our results above; this suggests that the effect size is smaller than in previous studies, but larger than *d* = 0.20. The present experiment lacked power to detect a true effect. The effect became statistically equivalent at a Cohen’s *d* of 0.39, suggesting a small-medium effect, where participants gave more in the public than the eyes conditions.

**Individual differences in SVO**

Given the limited sample size for egoists, we only conducted equivalence tests for private (combined Eyes and No Eyes conditions) and public conditions. We set the minimum effect size of interest to be Cohen’s d of 0.39, which was the smallest effect size of interest from Simpson & Willer (2008).

***Egoists (Public vs. Private).*** The effect observed is not statistically equivalent, because the larger of the two *p* values is more than .05, *t*(57.13) = 0.51, *p* = .308. This indicates that we cannot reject the hypothesis that the true effect is smaller than *d* = -0.39 or larger than *d* = 0.39 (see Figure S5). In other words, the conditions are not equivalent, but the present experiment lacked power to detect a true effect.

***Prosocials (Public vs. Private).*** The effect observed is not statistically equivalent, because the larger of the two *p* values is more than .05, *t*(73.56) = 1.48, *p* = .072, however it was trending. This indicates that we cannot reject the hypothesis that the true effect is smaller than *d* = -0.39 or larger than *d* = 0.39 (see Figure S5). In other words, the conditions are not equivalent, but the present experiment lacked power to detect a true effect.

Thus, these results are suggestive of an effect which is as or more extreme than *d* = 0.39, however the present study was underpowered to detect it.

**Table S3.2**

Factor loadings and component score coefficients of the three components from the post-experimental questionnaire identified by the PCA. Coefficients less than .100 were omitted.

| Question |  | | | Component | | | | | | |
| --- | --- | --- | --- | --- | --- | --- | --- | --- | --- | --- |
|  |  |  | | Factor Loadings | | | | Component Score Coefficient | | |
|  | *M* | | *SD* | 1 | 2 | 3 | 1 | | 2 | 3 |
| **How much did you think about the following points when you decided the amount you shared?** |  | |  |  |  |  |  | |  |  |
| 1. Allocation of more money to the recipient benefits me as well as the recipient. | 4.02 | | 1.69 | .241 | **.535** | .214 | .035 | | .264 | .154 |
| 1. Someone is watching the amount of money I allocate to the recipient. | 2.88 | | 1.79 | **.704** |  |  | .103 | | -.035 | -.072 |
| 1. I should give the amount the recipient expects | 3.20 | | 1.56 | **.561** | .453 | .101 | .082 | | .224 | .073 |
| 1. I only have to give as much money as I want to regardless of the recipient's desire. | 4.13 | | 1.64 | -.128 | **-.322** | .189 | -.019 | | -.159 | .137 |
| 1. The recipient will be angry if I allocate more money to myself than to him or her. | 3.49 | | 1.75 | .**609** | .242 | -.201 | .089 | | .120 | -.145 |
| 1. I should think of the recipient. | 4.97 | | 1.58 | .321 | **.740** | .227 | .047 | | .365 | .164 |
| 1. I will feel guilty if I don't share an equal amount with the recipient | 4.62 | | 1.93 | .405 | **.712** |  | .059 | | .351 | .051 |
| 1. Someone is counting how much money I allocate to the recipient | 3.16 | | 1.78 | **.739** |  | -.200 | .108 | | -.046 | -.144 |
| **How concerned were you about the following points when you decided the amount you shared?** |  | |  |  |  |  |  | |  |  |
| 1. Someone will see the amount of money I allocated and think I am a bad person. | 3.31 | | 1.83 | **.769** |  | -.360 | .112 | | -.001 | -.260 |
| 1. I may run into the recipient after the experiment | 2.52 | | 1.66 | **.706** |  | -.336 | .103 | | .008 | -.243 |
| 1. The experimenter will see the amount of money I allocated and form a poor opinion of me. | 2.74 | | 1.70 | **.725** |  | -.402 | .106 | | -.048 | -.291 |
| **How did you perceive the experimental situation?** |  | |  |  |  |  |  | |  |  |
| 1. A situation in which other people would find out how I behaved. | 3.76 | | 1.66 | **.695** | -.278 | .442 | .101 | | -.137 | .320 |
| 1. A situation in which other people would evaluate my behavior. | 4.38 | | 1.64 | **.669** | -.187 | . | .098 | | -.092 | .389 |
| 1. A situation in which other people would see my behavior. | 4.10 | | 1.70 | **.685** | -.282 | .463 | .100 | | -.139 | .335 |
| 1. A situation in which other people would know if I did something bad. | 3.36 | | 1.68 | **.802** | -.227 |  | .117 | | -.112 | -.023 |
| 1. A situation in which I should be concerned that other people were watching me. | 3.06 | | 1.68 | **.771** | -.217 | -.137 | .112 | | -.107 | -.099 |
| 1. A situation in which my good behavior would be evaluated by someone. | 3.98 | | 1.66 | **.732** |  | .147 | .107 | | -.038 | .106 |

**Table S3.3**

Regression of the three PCA components which predict the amount of money allocated to recipients in the dictator game, *F*(3,352) = 65.25, *p* < .001, *R* = .60, *R^2^* = .36.

|  | *B* | *SE* | *β* | *t* | *p* | Lower 95%CI of *B* | Upper 95%CI of *B* | Partial correlation |
| --- | --- | --- | --- | --- | --- | --- | --- | --- |
| Constant | 4.06 | .08 |  | 47.67 | < .001 | 3.90 | 4.23 |  |
| Component 1 | 0.20 | .08 | .10 | 2.33 | .020 | 0.03 | 0.36 | .10 |
| Component 2 | 1.11 | .09 | .56 | 13.00 | < .001 | 0.94 | 1.28 | .56 |
| Component 3 | 0.40 | .08 | .20 | 4.66 | < .001 | 0.23 | 0.56 | .20 |


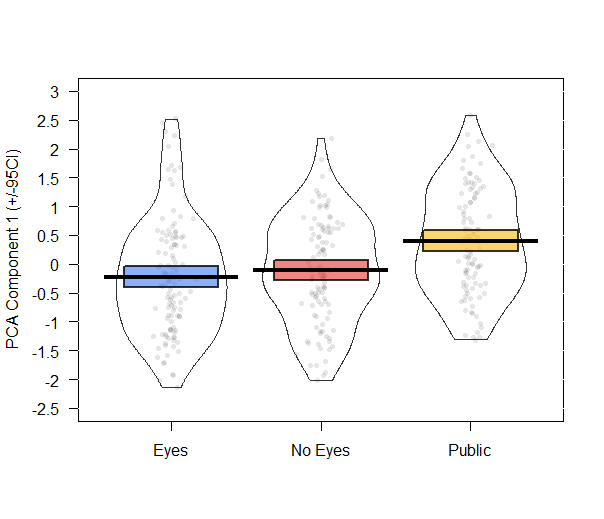


**Fig S1. Mean (+/- 95% CI) of the PCA Component 1 scores by experimental condition.** Dots are individual responses, with jitter so they don’t all overlap.


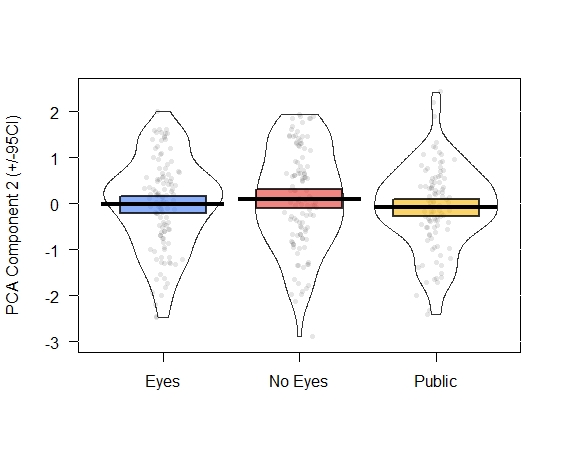


**Fig S2**. Mean (+/- 95% CI) of the PCA Component 2 scores by experimental condition. Dots are individual responses, with jitter so they don’t all overlap.


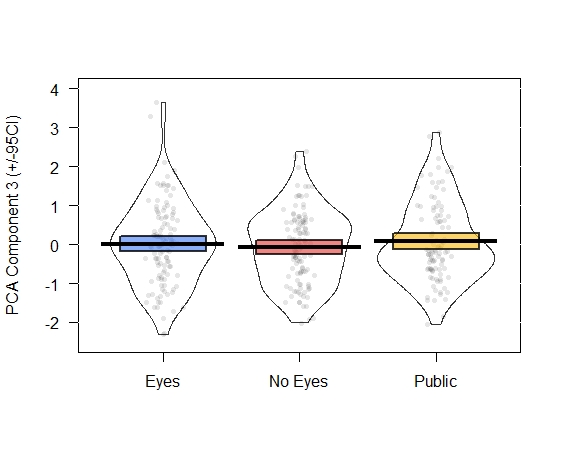


**Fig S3***.* **Mean (+/- 95% CI) of the PCA Component 3 scores by experimental condition.** Dots are individual responses, with jitter so they don’t all overlap


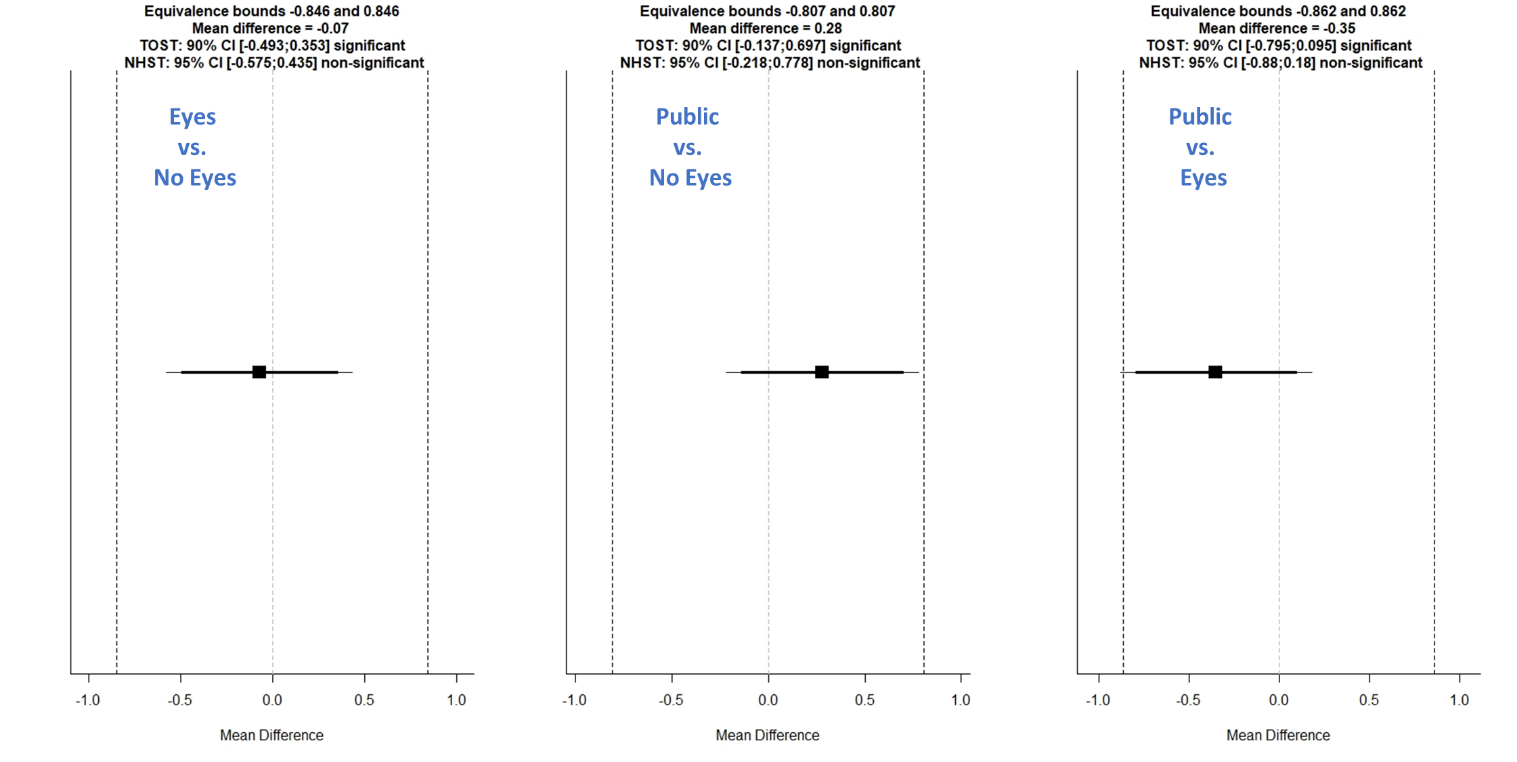


**Fig S4***.* **Results from the tests of equivalence (TOST) comparing the main effect of condition.** Comparison between Eyes and No Eyes conditions (left), Public and No Eyes conditions (middle), and Public and Eyes conditions (left). These results suggest that all conditions are equivalent, when comparing to an effect size of *d* = +/- 0.42. Note that the effects were not equivalent at smaller effect sizes (i.e., *d* of 0.20); the present student was underpowered to detect small effects.


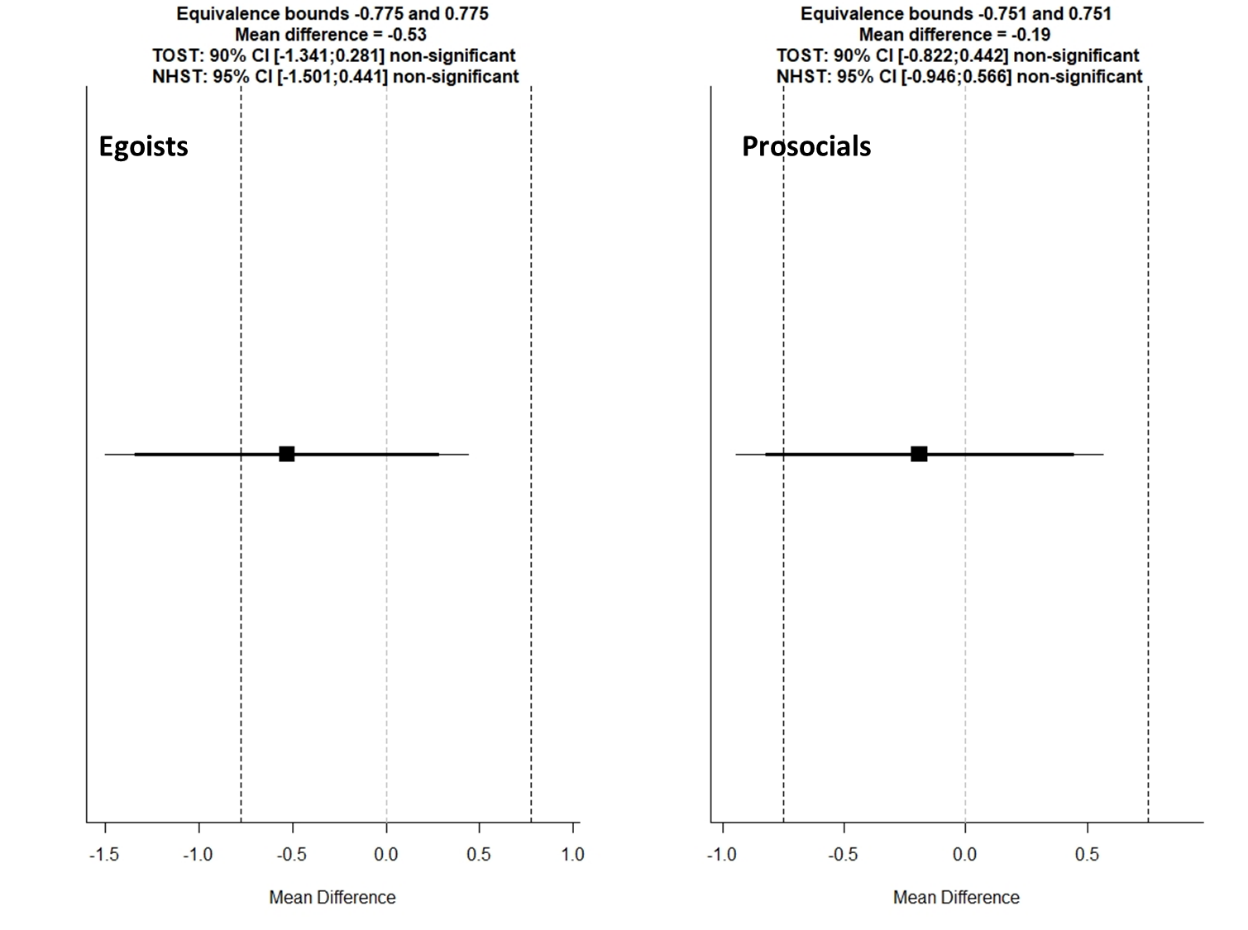


**Fig S5***.* **Results from the tests of equivalence (TOST) comparing egoists in the private and public conditions (left) and prosocials in the private and public conditions (right).** These results suggest that all conditions are not equivalent when comparing to an effect size of *d* = +/- 0.39. This study was underpowered to detect an effect of this size.
